# Loss-of-Function Mutations in the *CFH* Gene Affecting Alternatively Encoded Factor H-like 1 Protein Cause Dominant Early-Onset Macular Drusen

**DOI:** 10.1016/j.ophtha.2019.03.013

**Published:** 2019-10

**Authors:** Rachel L. Taylor, James A. Poulter, Susan M. Downes, Martin McKibbin, Kamron N. Khan, Chris F. Inglehearn, Andrew R. Webster, Alison J. Hardcastle, Michel Michaelides, Paul N. Bishop, Simon J. Clark, Graeme C. Black, United Kingdom Inherited Retinal Dystrophy Consortium, Graeme Black, Georgina Hall, Stuart Ingram, Rachel Taylor, Forbes Manson, Panagiotis Sergouniotis, Andrew Webster, Alison Hardcastle, Michel Michaelides, Vincent Plagnol, Nikolas Pontikos, Michael Cheetham, Gavin Arno, Alessia Fiorentino, Chris Inglehearn, Carmel Toomes, Manir Ali, Martin McKibbin, Claire Smith, Kamron Khan, Susan Downes, Jing Yu, Stephanie Halford, Suzanne Broadgate, Veronica van Heyningen

**Affiliations:** 1 Division of Evolution and Genomic Sciences, School of Biological Sciences, Faculty of Biology, Medicine and Health, Manchester Academic Health Science Centre, University of Manchester, Manchester, United Kingdom; 2 Manchester Centre for Genomic Medicine, Central Manchester University Hospitals NHS Foundation Trust, Manchester Academic Health Science Centre, St. Mary’s Hospital, Manchester, United Kingdom; 3 Section of Ophthalmology and Neuroscience, Leeds Institute of Medical Research, University of Leeds, Leeds, United Kingdom; 4 Oxford Eye Hospital, Oxford University Hospitals, NHS Foundation Trust, Oxford, United Kingdom; 5 Nuffield Department of Clinical Neuroscience, John Radcliffe Hospital, Oxford, United Kingdom; 6 Department of Ophthalmology, St. James’s University Hospital, Leeds, United Kingdom; 7 UCL Institute of Ophthalmology, University College London, London, United Kingdom; 8 Moorfields Eye Hospital, London, United Kingdom; 9 Manchester Royal Eye Hospital, Central Manchester University Hospitals NHS Foundation Trust, Manchester Academic Health Science Centre, Manchester, United Kingdom; 10 The Lydia Becker Institute of Immunology and Inflammation, Faculty of Biology, Medicine and Health, University of Manchester, Manchester, United Kingdom

**Keywords:** AMD, age-related macular degeneration, AP, alternative pathway, BrM, Bruch’s membrane, CCP, complement control protein, CFH, complement factor H, ECM, extracellular matrix, EOMD, early-onset macular drusen, FH, factor H, FHL-1, factor H like-1, MAF, minor allele frequency

## Abstract

**Purpose:**

To characterize the molecular mechanism underpinning early-onset macular drusen (EOMD), a phenotypically severe subtype of age-related macular degeneration (AMD), in a subgroup of patients.

**Design:**

Multicenter case series, in vitro experimentation, and retrospective analysis of previously reported variants.

**Participants:**

Seven families with apparently autosomal dominant EOMD.

**Methods:**

Patients underwent a comprehensive ophthalmic assessment. Affected individuals from families A, B, and E underwent whole exome sequencing. The probands from families C, D, F, and G underwent Sanger sequencing analysis of the complement factor H (*CFH*) gene. Mutant recombinant factor H like-1 (FHL-1) proteins were expressed in HEK293 cells to assess the impact on FHL-1 expression and function. Previously reported EOMD-causing variants in *CFH* were reviewed.

**Main Outcome Measures:**

Detailed clinical phenotypes, genomic findings, in vitro characterization of mutation effect on protein function, and postulation of the pathomechanism underpinning EOMD.

**Results:**

All affected participants demonstrated bilateral drusen. The earliest reported age of onset was 16 years (median, 46 years). Ultra-rare (minor allele frequency [MAF], ≤0.0001) *CFH* variants were identified as the cause of disease in each family: *CFH* c.1243del, p.(Ala415ProfsTer39) het; c.350+1G→T het; c.619+1G→A het, c.380G→A, p.(Arg127His) het; c.694C→T p.(Arg232Ter) het (identified in 2 unrelated families in this cohort); and c.1291T→A, p.(Cys431Ser). All mutations affect complement control protein domains 2 through 7, and thus are predicted to impact both FHL-1, the predominant isoform in Bruch’s membrane (BrM) of the macula, and factor H (FH). In vitro analysis of recombinant proteins FHL-1_R127H_, FHL-1_A415f/s_, and FHL-1_C431S_ demonstrated that they are not secreted, and thus are loss-of-function proteins. Review of 29 previously reported EOMD-causing mutations found that 75.8% (22/29) impact FHL-1 and FH. In total, 86.2% (25/29) of EOMD-associated variants cause haploinsufficiency of FH or FHL-1.

**Conclusions:**

Early-onset macular drusen is an underrecognized, phenotypically severe subtype of AMD. We propose that haploinsufficiency of FHL-1, the main regulator of the complement pathway in BrM, where drusen develop, is an important mechanism underpinning the development of EOMD in a number of cases. Understanding the molecular basis of EOMD will shed light on AMD pathogenesis given their pathologic similarities.

Age-related macular degeneration (AMD) represents a leading cause of irreversible vision loss, accounting for 8.7% of global blindness.^1^ The condition is characterized by inflammation and the deposition of extracellular material, in the form of drusen, in Bruch’s membrane (BrM). Drusen can cause metabolic disruption that leads to dysfunction and death of retinal pigment epithelium. Later stages of AMD may be characterized by geographic atrophy or choroidal neovascularization; these are associated with severe loss of central vision. It is now widely accepted that an excessive inflammatory response driven by inadequate regulation of the complement cascade is a major contributory factor to AMD.^2^ Age-related macular degeneration is a disease of multifactorial cause with a strong genetic component, and the role of the complement pathway in AMD pathogenesis is corroborated by the implication of genetic variants in a number of complement factors with AMD risk.^3^, ^4^ Genetic variants associated with AMD represent a broad allelic range, from common polymorphisms (minor allele frequency [MAF], >1%) that confer a relatively low risk of disease^5^, ^6^, ^7^, ^8^, ^9^ to relatively rare variants (MAF, <1%) that demonstrate high penetrance, for example, the p.(Arg1210Cys) substitution in complement factor H (*CFH*).^10^

The complement system is a crucial component of host innate immunity.^11^ It is a cascade system comprising 3 activation pathways—classical, lectin, and alternative—each of which is engaged uniquely, but that converge on 3 common goals: modifying the membrane of an unwanted cell for phagocyte recognition, generation of membrane attack complexes for cell lysis, and promoting an inflammatory response.^2^ Complement can activate on all surfaces, both host or foreign, so the host requires mechanisms to prevent inappropriate self-directed damage.^12^ The alternative pathway (AP) is constantly active at a low level and contains a positive feedback loop to allow rapid amplification of the complement response. Tight regulation is required to maintain balanced immune homeostasis, and it is increasingly recognized that defective regulation of the AP plays a central role in human disease.^13^, ^14^

The *CFH* gene encodes a 155-kDa plasma protein known as factor H (FH)^15^ that functions as a complement regulator by binding to host surfaces to protect them against complement activation. It mainly exerts its effect on the AP, negatively regulating the positive feedback loop. Factor H harbors 20 complement control protein (CCP) domains, each comprising 60 amino acids, that can be grouped into 3 functional domains: CCP domains 1 through 4 form a binding site for cofactor activity, whereas both CCP domains 6 through 8 and CCP domains 19 and 20 facilitate binding of FH via glycosaminoglycans to cell surfaces and extracellular matrices (ECMs).^2^, ^16^

Disease-causing variants in *CFH* result in 3 distinct pathologic syndromes: atypical hemolytic uremic syndrome, C3 glomerulopathy (a clinical entity that encompasses C3 glomerulonephritis and dense deposit disease, formerly membranoproliferative glomerulonephronophthitis type II), and AMD. An apparent genotype–phenotype correlation exists whereby most atypical hemolytic uremic syndrome–causing variants affect CCP domains 19 and 20,^17^ whereas variants associated with AMD predominantly affect CCPs 1 through 4 and 6 through 8.^18^, ^19^ Boon et al^20^ in 2008 and van de Ven et al^21^ in 2012 showed that an early-onset subtype of AMD was caused by monogenic inheritance of ultrarare variants in *CFH*. This AMD subtype (which for clarity we term *early-onset macular drusen* [EOMD]) demonstrates a much earlier age of onset (mean age, 50 years), causing many more years of substantial visual loss than AMD.^20^, ^21^ Few EOMD cases have been reported since.^22^, ^23^, ^24^, ^25^

Alternative splicing of *CFH* exon 9 produces a variant of FH known as factor H like 1 (FHL-1) that is identical to CCPs 1 through 7 of FH, has a unique carboxy-terminal tail of 4 amino acids, and is significantly smaller (49 kDa). Recent work has suggested that FHL-1 retains the same regulatory functions as FH and is able to bind to surfaces via its single glycosaminoglycan-interaction domain at CCP domains 6 and 7 to regulate the complement cascade.^26^ Impaired FH and FHL-1 function leads to disease as a result of inflammation and cellular debris mishandling because of excessive AP activation driven by defective regulation of the complement cascade.^2^, ^26^ Genetic studies of AMD fail to distinguish between FH and FHL-1. Factor H-like 1 is the major isoform present within BrM of the retina,^26^, ^27^ a major site of AMD pathogenesis and where drusen, the characteristic lesions of AMD, form. It has been suggested that it is this isoform that protects BrM against complement activation.^26^ In this study, we performed functional and variant localization analysis of the EOMD-causing variants that lie within FHL-1 and defined the mechanism responsible for a subgroup of patients affected by this phenotypically severe condition.

## Methods

### Ethics and Patient Recruitment

Ethics committee approval was obtained from the North West Regional Ethics Committee for all aspects of this study (identifier, 15/YH/0365), and the protocol observed the tenets of the Declaration of Helsinki. Written informed consent was obtained from each study participant as an essential prerequisite for study inclusion. Patients with EOMD were included in our analysis. Given the heterogeneous nature of drusen and to limit bias, we did not select patients based on the type or size of drusen they demonstrated.

### Clinical Assessment

Each patient underwent full ophthalmic assessment including visual acuity and dilated fundus examination. Fundus photographs were obtained using Ultra-Widefield Optos color fundus imaging (Optos plc, Dunfermlin, UK). Fundus autofluorescence imaging was conducted using either the Spectralis (Heidelberg Engineering Ltd, Heidelberg, Germany) or ultra-widefield confocal scanning laser imaging (Optos plc). OCT was performed using the Spectralis OCT platform (Heidelberg Engineering). Electroretinography was performed to standards specified by the International Society for Clinical Electrophysiology of Vision. Clinical history also was obtained to discern the presence of additional health problems.

### Whole-Exome Sequencing

Whole-exome sequencing, performed as outlined in a previous publication,^28^ was carried out as part of an ongoing study on inherited retinal disease (United Kingdom Inherited Retinal Dystrophy Consortium) in families without a molecular diagnosis after next generation sequencing (NGS) screening for a panel of 105 or 176 genes known to cause inherited retinal dystrophy. Detailed methodology can be found in Supplemental Data A (available at www.aaojournal.org). Whole-exome sequencing data from affected relatives (A:II.1 and A:II.3; E:III.11 and E:II.8) was analyzed for shared rare variants. Variants in genes known to be involved in inherited retinal disease were examined as a priority. Identified variants were interpreted according to the Association for Clinical Genetic Science Best Practice Guidelines for Variant Classification 2018.

### Sanger Sequencing of *CFH*

Seventy-five patients diagnosed with dominant or early drusen whose results were negative for the EFEMP1 c.1033C→T; p.(Arg345Trp) variant were identified retrospectively from a referrals database and were subjected to screening for variants in the *CFH* gene by Sanger sequencing. The coding exons and flanking intronic sequences of *CFH* (NM_000186) plus an additional 4 amino acids unique to the alternative transcript FHL-1 (exon 10, NM_0010149975) were amplified by polymerase chain reaction and subject to bidirectional Sanger sequencing (see Supplemental Data A for details). Variants were interpreted as before (see “Whole-Exome Sequencing”).

### Functional Characterization of Genetic Variants

N-terminal His-tagged DNA sequences encoding human FHL-1 or mutant versions of FHL-1 were synthesized and sub-cloned in to pcDNA3.1 by GeneArt (Invitrogen; Carlsbad, CA). Plasmid DNA was transfected into HEK293 cells using polyethylenimine, and culture media was harvested from transfected cells at 24, 48, 72, and 144 hours. His-tagged recombinant proteins were purified from the harvested media using Amintra Ni-NTA affinity resin (Expedeon, Cambridge, UK) by gravity flow chromatography. Cell lysates were made from transfected HEK293 cells and quantified using the Pierce BCA assay (Thermofisher Scientific, Waltham, MA). Western blotting was conducted as previously described.^26^ Detailed methodologies for plasmid preparation, transformation and transfection, purification of recombinant proteins, and Western blotting can be found in Supplemental Data A.

## Results

### Retinal Findings

**Figure 3:**
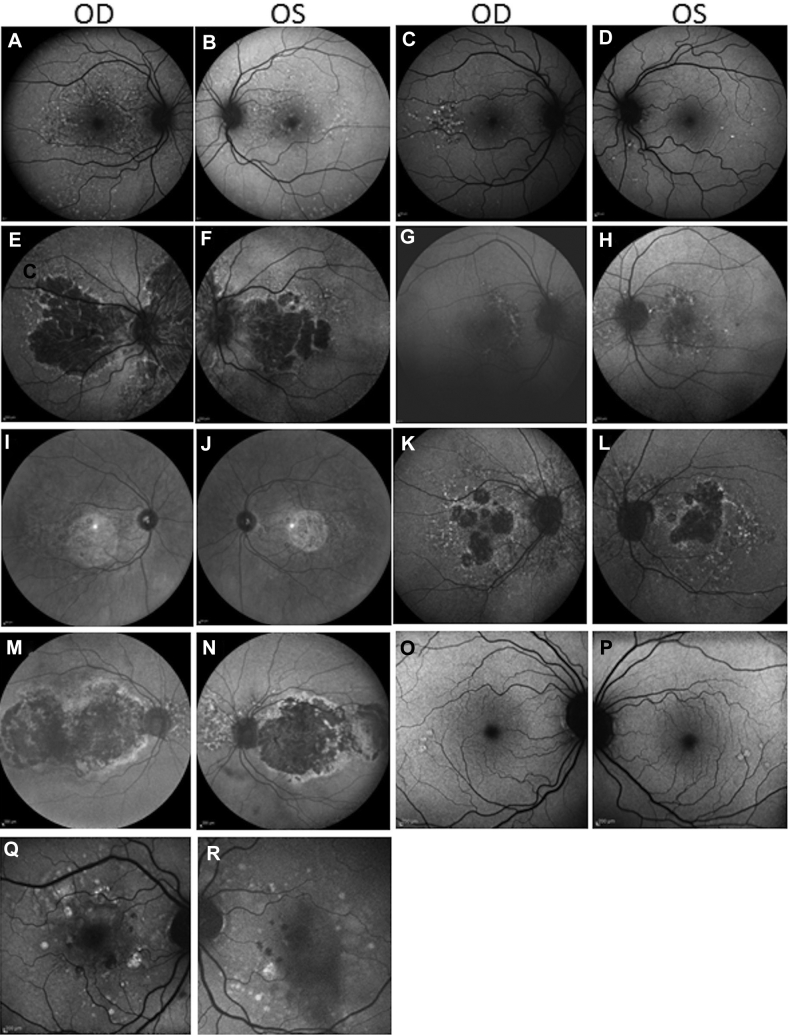
Fundus autofluorescence imaging in patients with early-onset macular drusen with rare *CFH* variants. **A**, **B**, Patient A:II.3, 51 years of age, showing drusen at the macula and extending beyond the vascular arcades. **C**, **D**, Patient A:II.1, 49 years of age, with drusen extending outside the macular region (**C**). **E**, **F**, Patient B:II.2, 89 years of age, showing drusen and retinal atrophy at the macula and in the nasal retina in both eyes. **G**, **H**, Patient B:I.1, 61 years of age, with bilateral macular drusen and drusen nasal to the optic discs. **I**, **J**, Patient C:I.2, 64 years of age, showing loss of central signal consistent atrophy; drusen are present around the atrophy and optic nerve. **K**, **L**, Patient D:I.7, 26 years of age, showing geographic atrophy and large colloidal macular drusen. **M**, **N**, Patient E:II.2, 53 years of age, showing hypoautofluorescence centrally resulting from geographic atrophy with a surrounding ring of hyperautofluorescence and drusen nasal to the optic disc. **O**, **P**, Patient F:II.2, 54 years of age, showing sparse temporal drusen. **Q**, **R**, Patient G:III.7, 66 years of age, showing scattered macular drusen and patches of geographic atrophy.

We report 10 individuals from 7 unrelated families with a monogenic form of EOMD (Fig S1, available at www.aaojournal.org). Retinal phenotypes are summarized below and in Table 1. Detailed ophthalmic histories for each participant can be found in Supplemental Data B (available at www.aaojournal.org). None of the individuals included in this study showed evidence of renal disease. The median age of drusen onset was 46 years. The average age at onset in our cohort is skewed by late identification of disease in individual B:II.2, who did not receive a diagnosis until 80 years of age. The earliest documented age at which drusen were identified was 16 years in individual A:II.1, younger than has been reported previously in EOMD. In all affected study participants, drusen were bilateral and broadly symmetrical and were visible on fundus examination or color photographs (Fig S2, available at www.aaojournal.org), fundus autofluorescence images (Fig 3), OCT images (Fig S4, available at www.aaojournal.org), or a combination thereof. The impact on visual acuity was varied, from no apparent impact (B:I.1) to mild (A:II.3, A:II.1, C:I.2, D:I.7, E:III.11, F:II.2, and G:III.7), moderate (D:II:2 and E:II.2), and severe (B:II.2) visual loss. As shown in Figures S2 through S4, varying phenotypes and degrees of disease severity were present in study participants. This possibly reflects that individuals in our cohort represent a range of ages and thus various disease stages. However, in younger participants, we cannot rule out nonprogressive disease at this time.

**Table 1:** Phenotypic and Genetic Findings in Early-Onset Macular Drusen Patients with *CFH* Mutations

Study Identification∗	Gender	Age (yrs)		Family History†	Phenotype	Snellen Visual Acuity (logMAR)		Electrophysiology Findings	Mutation	Complement Control Protein Domain	GnomAD Allele Count (Allele Frequency)
		Age at onset∗	At Most Recent Examination			Right Eye	Left Eye				
A:II.3	M	18	51	Yes	Bilateral, scattered or widespread early-onset drusen, RPE mottling at the fovea, reduced VA.	+5.75 (0.22; 6/9^–1^)	+3.75 (0.0; 6/6)	Normal and grossly symmetrical light-adapted response. Slightly reduced dark-adapted response in right eye suggesting asymmetrical rod involvement. Normal EOG results.	c.1243del, p.(Ala415Profs∗39) het	7	—
A:II.1	M	16	49		Bilateral widespread drusen, concentrated temporal to and within the macula.	6/3.8^–1^	6/3.8^–1^	Normal and grossly symmetrical light-adapted response. Slightly reduced dark-adapted response in left eye suggesting asymmetrical rod involvement. Normal EOG results.			
B:II.2	F	80	89	Yes	Bilateral, symmetrical, outer retinal atrophy, multiple drusen, severe visual loss.	—	—	NA	c.350+1G→T het	2	1/245972 (0.000004066)
B:I.1	M	61	61		Multiple drusen bilaterally, pattern similar to that seen in affected mother (B.II.1).	6/6	6/6	NA			
C:I.2	F	54	64	Yes	Bilateral drusen surrounding central atrophy and extending into the arcades. Patchy atrophy in the peripheral retina with reticular and drusenoid features.	6/6	6/15	NA	c.694C→T p.(Arg232Ter) het‡	4	2/244650 (0.000008175)
D:I.7	F	26	50	Yes	Large “colloid” drusen.	6/5	6/5	NA	c.694C→T p.(Arg232Ter) het‡	4	2/244650 (0.000008175)
D:II.2	M	50	64		Bilateral retinal drusenoid dystrophy with CNV and significant scarring.	1/60	6/24	NA			
E:II.2	F	50	52	Yes	Early-onset macula dystrophy, macular and midperipheral drusen.	6/120 (1.34)	6/96 (1.24)	Extinguished PERGs, normal EOG results, normal ERG results; Ishihara: 1/17 right eye, 2/17 left eye	c.1291T→A, p.(Cys431Ser) het‡	6	1/245702 (0.000004070)
E:III.2	M	40	53		Bilateral small, sparse drusen at the maculae.	6/4	6/7.5	NA			
F:II.2	F	46	54	Yes	Isolated sparse drusen within the macula and temporal raphes.	6/6 (–4/–1.00 × 180)	6/4.8 (–4.50/–1.00 × 170)	NA	c.380G→A, p.(Arg127His) het‡	2	2/121206 (0.0000165)
G:III.7	F	45	66	Yes	Bilateral large, sparse white/yellow drusen at the maculae, nasal to the disc and the surrounding arcades. Patchy geographic atrophy in the left eye.	6/6 (+2.75/–1.00 × 17)	6/6 (+2.75/–1.50 × 165)	NA	c.619+1G→A het	3–4	—

CNV = choroidal neovascularization; EDT = electrodiagnostic testing; EOG = electrooculogram; ERG = electroretinogram; F = female; M = male; NA = not available; PED = pigment epithelial detachment; PERG = pattern electroretinogram; RPE = retinal pigment epithelium; VA = visual acuity; — = measurement not possible.

∗ Age at onset was defined as age at which retinal changes were detected first.

† A positive family history was defined as another blood relative reported to be affected by macular disease or drusen.

‡ Mutation previously reported as disease causing.

In family A, both affected siblings demonstrated small drusen, scattered throughout the retina in an appearance typical of basal laminar drusen (i.e., so-called stars-in-the-sky appearance). Ophthalmic examination of their mother found that she was unaffected. Proband B:I.1 from family B was found to have multiple small drusen at the macula and nasal to the optic discs. His mother, B:II.2, was more advanced, showing drusen as well as atrophy at both the macula and the nasal retina. Participant C:I.1 demonstrated central scotomata and drusen surrounding a region of central atrophy that extended to the vascular arcades. She showed patchy atrophy with reticular and drusenoid features in the retinal periphery with midperipheral sparing. Her mother and sister also had received a diagnosis of macular drusen. The proband from family D (D:I.7) demonstrated multiple large drusen bilaterally associated with atrophy of the right macula. Her affected father (D:II.2) demonstrated advanced neovascular macular degeneration. Family history revealed the proband’s paternal aunt (D:II.1) and great grandfather (D.IV.6) had experienced visual deterioration in their forties. It was also noted that her paternal great aunt (D.IV.2) and great uncle (D:IV.5) were affected in their fifties, although no further information was available. Her paternal grandmother (D:III.3) died at 56 years of age, and it is not known whether she also was affected. The proband from family E (E:III.11) demonstrated central vision problems. Fundus autofluorescence revealed hypoautofluorescence centrally resulting from geographic atrophy with a surrounding ring of hyperautofluorescence and drusen nasal to the optic disc. Pigmentary changes and drusen also were seen in the peripheral retina. Her affected son (E:II.8) was found to have small, sparse macular drusen at 40 years of age. Family history revealed multiple affected members in family E. Funduscopy of the proband from family F (F:II.2) revealed isolated sparse drusen within the temporal macula. At the time of her diagnosis, her mother (F:III.2) was being treated for choroidal neovascularization. Proband G:III.7 from family G demonstrated clustered drusen spread throughout the macula, with early nonfoveal geographic atrophy in the left eye. Her sister (G:III.3) and 2 brothers (G:III.1 and G.III.4) also were affected. Her mother died at 30 years of age and her father died at 80 years of age with no known visual problems.

### Genetic Findings

The probands from families A, B, and E underwent testing for variants in the coding and flanking intronic regions of 105 or 176 known retinal dystrophy-causing genes, including a number of genes associated with macular drusen (*ABCA4*, NM_000350; *CA4*, NM_000717; *CNGB3*, NM_019098; *EFEMP1*, NM_001039348; *PROM1*, NM_006017; and *TIMP3*, NM_000362). No putative disease-causing or carrier variants were identified. Subsequently, each was recruited to the United Kingdom Inherited Retinal Dystrophy Consortium study, and whole-exome sequencing was conducted on DNA from the affected sibling pair of family A (A:II.1 and A:II.3), the proband of family B (B:II.2), and the affected mother (E.II.2) and son (E.I.1) of family E. In each family, an ultrarare (MAF, <0.0001) or novel heterozygous *CFH* variant was identified as the probable cause of disease (Table 1). NGS analysis and interpretation of variants can be found in Supplemental Data C (available at www.aaojournal.org).

A cohort of 75 patients diagnosed with macular drusen and demonstrating negative results for the *EFEMP1* c.1033C→T p.(Arg345Trp) variant were screened for variants in the coding and flanking intron regions of the *CFH* gene (NM_000186), including an alternatively encoded exon from the transcript NM_0010149975. Four patients (the probands of families C, D, F, and G: C:I.2, D:I.7, F:II.2, and G:III.7) were found to harbor novel or ultrarare (MAF, <0.0001) heterozygous variants in the *CFH* gene (Table 1).

**Figure 5:**
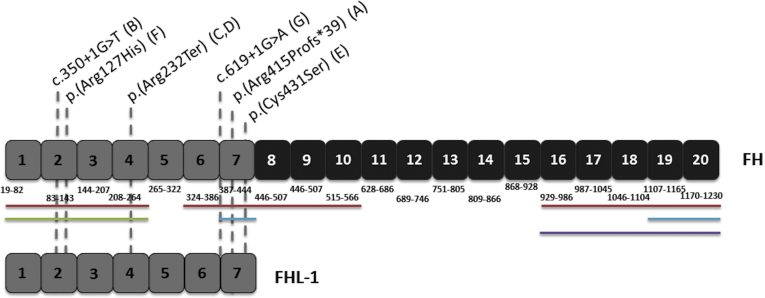
Protein schematic of factor H (FH) and factor H like-1 (FHL-1) showing protein domains and corresponding amino acid positions with locations of mutations identified in our early-onset macular drusen cohort. Factor H contains 20 complement control protein (CCP) domains (top), whereas FHL-1 encodes 7 CCP domains (bottom), identical to FH CPP domains 1 through 7. Regions associated with C3b binding are indicated by adjacent red bars, cofactor activity is indicated by the green bar, heparin binding sites are indicated by blue bars, and the sialic acid binding site is indicated by the purple bar. The locations of mutations identified by this study are indicated by dashed grey lines. The parenthetical letter after the mutation nomenclature indicates the study identification of the family in which the mutation was identified.

In total, 6 different *CFH* variants were identified in 7 EOMD families (Table 1). Segregation analysis was performed where possible, and the respective variant was segregated with disease in each case (family members tested, their disease status, and their genetic status are indicated in Fig S1). Three mutations that have not been reported previously in association with disease represent novel EOMD-causing mutations: *CFH* c.1243del, p.(Ala415ProfsTer39) het; *CFH* c.350+1G→T het; and *CFH* c.619+1G→A het. The 3 remaining variants have been reported previously as disease causing: *CFH* c.380G→A, p.(Arg127His)^25^, ^29^; *CFH* c.694C→T, p.(Arg232Ter)^30^ identified in 2 unrelated families in this cohort; and *CFH* c.1291T→A, p.(Cys431Ser), which has been identified in the homozygous state underlying membranoproliferative glomerulonephronophthitis type I^31^ and dense deposit disease.^30^ All 6 mutations identified in this study affect CCP domains 2 through 7 (Table 1) and therefore are predicted to impact both FH and FHL-1 (Fig 5).

### Functional Characterization of *CFH* Mutations in Recombinant Factor H-like 1

Previous studies have shown that rare *CFH* variants underlying atypical hemolytic uremic syndrome and EOMD can prevent or delay secretion of FH^32^, ^33^, ^34^, ^35^ or can impair protein function severely, causing reduced FH activity^22^, ^25^, ^33^, ^36^ leading to dysregulation of the complement pathway. However, the impact of rare *CFH* variants on the function of the alternative isoform FHL-1 has not been investigated previously. Given the probable importance of FHL-1 in the EOMD phenotype,^26^ we investigated the expression and secretion of mutant forms of FHL-1 containing the respective mutations identified in EOMD families A (FHL-1_A415f/s_), E (FHL-1_C431S_), and F (FHL-1_R127H_) compared with full-length wild-type FHL-1 (FHL-1_402Y_). Secreted His-tagged recombinant protein was purified from media by affinity chromatography, and lysates were made from transfected cells for analysis of intracellular expression of recombinant wild-type or mutant FHL-1.

**Figure 6:**
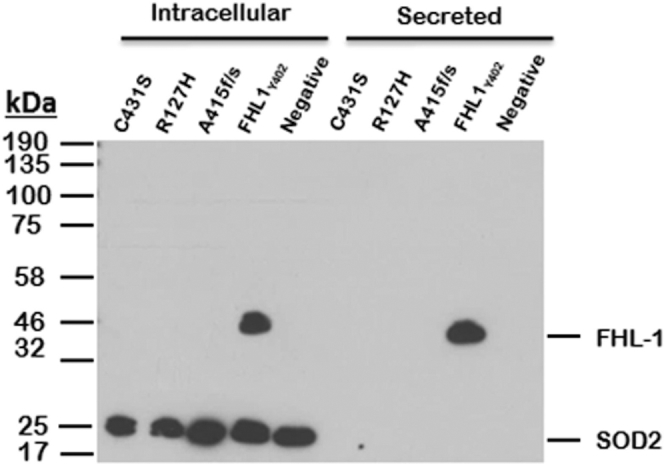
Expression of wild-type and mutant factor H like-1 (FHL-1) in transfected HEK293 cells. HEK293 cells were transfected stably with His-tagged wild-type pcDNA3.1-FHL-1 (FHL-1_Y402_) or 1 of 3 mutant constructs (FHL-1_C431S_, FHL-1_R127H_, or FHL-1_A415f/s_), as indicated at the top of the gel image, to assess the effect of the identified variants on protein expression and secretion over 144 hours. Recombinant proteins purified from culture media (secreted) and cell lysates (intracellular) were subjected to sodium dodecyl sulfate–polyacrylamide gel electrophoresis (SDS-PAGE) and transferred to a nitrocellulose membrane. The figure shows Western blot results from analysis of experimental samples for OX23. Presence of a band indicates presence of recombinant FHL-1. Cells transfected with mutants did not secrete a detectable FHL-1 product (approximately 51 kDa) in comparison with wild-type FHL-1. Mock (no DNA) transfected cells did not produce an FHL-1 product, as expected. Analysis of cell lysates found no accumulation of mutant recombinant proteins intracellularly, but wild-type recombinant FHL-1 was found to be present. SOD2 (approximately 26 kDa) indicates equal sample loading of cell lysates. OD = right eye; OS = left eye.

Western blotting for the recombinant proteins revealed that FHL-1_402Y_ (wild-type) transfected cells secreted a protein product of expected size (51 kDa, slightly larger than native FHL-1 because of its N-terminal His-tag modification). However, cells transfected with mutant plasmids did not secrete a 51-kDa protein product at detectable levels within 144 hours of transfection (Fig 6). Mock (i.e., no vector) transfected cells did not secrete any detectable protein product, as expected. Next, we investigated whether the mutant proteins were being expressed but retained intracellularly by examining lysates made from the transfected cells. Western blot for intracellular expression recombinant mutant and wildtype proteins indicated mutant proteins were not detectable when analyzed using OX23 (Abcam, Cambridge, UK). In comparison, FHL-1_402Y_ (wildtype) was present in abundance as indicated by the intense approximately 51-kDa band despite equal protein loading as indicated by SOD2 (approximately 26 kDa). These findings suggest that although wildtype recombinant FHL-1 is expressed and secreted, mutant forms of FHL-1 are not. The absence of mutant proteins intracellularly suggests they are degraded rapidly by the cell soon after synthesis, possibly because they are unfolded or unstable, rather than synthesized and accumulated within the cell because they cannot be secreted.

Our findings indicate that the c.1243del, p.(Ala415ProfsTer39) het; c.1291T→A, p.(Cys431Ser) het; and c.380G→A, p.(Arg127His) het variants found to underlie disease in families A, E, and F, respectively, consequently could be considered to result from loss of function of FHL-1, because the mutant proteins may be degraded rapidly on synthesis, not secreted by the cell, or both. Although beyond the scope of this study, it may be possible to assess this via the application of inhibitors of degradation pathways. The remaining putative EOMD-causing variants—c.350+1G→T het (family B), c.694C→T p.(Arg232Ter) het (families C and D), and c.619+1G→A het (family G)—also are predicted to be loss-of-function variants. This leads us to suggest that haploinsufficiency of FHL-1 is the pathologic mechanism underlying this severe, dominant EOMD phenotype in a subset of patients (i.e., the identified variants result in reduction in the amount of functional FHL-1 protein produced, which is not sufficient to support the normal function of the retinal pigment epithelium, leading to disease).

### Review of Previously Reported Early-Onset Macular Drusen Mutations

Following from this hypothesis, we reviewed previously reported cases of EOMD to define further their mutational mechanisms. For this analysis, we considered literature reports of mutations found to cause “basal laminar drusen,”^20^, ^21^ “cuticular drusen,”^23^, ^37^ and “early-onset AMD”^22^, ^24^, ^25^ in the absence of kidney disease. A total of 25 different variants in 29 families have been reported (Supplemental Data D, available at www.aaojournal.org). Of these, 19 are nonsynonymous missense variants. Three variants—c.1198C→A; p.(Gln400Lys) het, c.2850G→C; p.(Gln950His) het, and c.3628C→T; p.(Arg1210Cys) het—each have been identified in 2 unrelated families.^23^ Six different loss-of-function (i.e., splice-altering, frameshift, or nonsense) variants also have been reported. One nonsense variant (c.1222C→T, p.Gln408Ter het) has been identified in 2 unrelated families.^20^ Of the 29 reported EOMD cases, 75.8% (n = 22) are the result of variants affecting CCP domains 1 through 7 and impact both FH and FHL-1. Overall, including the cases reported here, 80.5% (n = 29) of reported EOMD-causing variants (n = 36) affect CCP domains 1 through 7 and thus impact FHL-1 and FH. Furthermore, 100% of previously reported putative loss-of-function variants (i.e., nonsense [n = 2], frameshift [n = 2], and splice-altering [n = 3]) causing EOMD affect CCP domains 1 through 7. Of the 15 missense variants affecting CCP domains 1 through 7, 71.3% (n = 11) have evidence that they prevent secretion or severely reduce protein function. The remaining 4 variants have not been assessed. In total, 81.8% (n = 18) of previously reported EOMD variants affecting CCP domains 1 through 7 likely result in haploinsufficiency of FHL-1 or FH. Taking into account the variants reported herein, this figure increases to 86.2% (n = 25). Thus, there is considerable evidence in support of the hypothesis that haploinsufficiency of FHL-1 is the molecular mechanism underpinning the development of this severe, early-onset phenotype in a proportion of cases. Two of the other reported missense variants (p.(Pr0139Ala) and p.(Arg175Gln)) have supporting evidence for their pathogenicity in that a different amino acid change at the same position of the peptide has been reported to cause *CFH*-related disease with a demonstrated effect on FH serum levels, suggesting that the mutant protein is not secreted or is degraded on synthesis or secretion.^25^, ^38^

## Discussion

This study identified unrelated families with a history of EOMD associated with deleterious ultrarare (MAF, ≤0.0001) heterozygous *CFH* pathogenic variants. It provided evidence that clinical signs begin significantly earlier than seen in typical AMD cases; the earliest reported onset of retinal changes in our cohort is 16 years of age. The 6 different variants identified (Table 1) all localized within CCP domains 2 through 7 and consequently impact FHL-1 and FH. Factor H-like 1 is a short form of FH that retains all of the regulatory functions of the full-length protein that has been suggested to be the predominant regulator of complement in BrM, the ECM layer that lies beneath the human retina, and the intercapillary septa.^26^ We propose that lost or reduced function of FHL-1 leads to dysregulation of complement turnover in the ECM of BrM and choriocapillaris and may be the mechanism driving development of EOMD in these cases; complement overactivation is thought to be an important driver of pathogenesis in early AMD.^39^

The identified putative splice-altering and nonsense variants are likely to result in haploinsufficiency of both FH and FHL-1. By contrast, the c.1243del mutation in exon 9 of *CFH* is predicted to have differing effects on the 2 isoforms. In FH (NM_000186), this frameshift is predicted to undergo nonsense-mediated decay (NMD) whereas in FHL-1 (NM_001014975), this same single base pair deletion is predicted to result in a frameshift within the penultimate exon that likely is to escape NMD, creating a stop codon 47 amino acid downstream (p.(Ala415ProfsTer47)) and resulting in an extension of the normal 449-amino acid product by 13 amino acids, as well as loss of its unique 4-amino acid C-terminal tail, which is known to have a role in the proteins binding to proinflammatory monomeric C-reactive protein.^40^ Predicting the impact of missense variants on protein function is challenging, particularly for the *CFH* gene.^33^ Both of the missense variants, p.(Cys431Ser) and p.(Arg127His), as well as the p.(Ala415ProfsTer47) frameshift variant, were investigated in vitro. By expressing these recombinant mutant FHL-1 proteins, we have demonstrated that none of the 3 allowed the production of a secreted FHL-1 product. We suggest that all 3 variants also may result in loss of protein function. The *CFH* p.(Cys431Ser) variant replaces the highly conserved, third cysteine residue of the seventh CCP domain. One of the defining characteristics of a CCP domain is the presence of 2 disulphide bonds with the cysteine–cysteine pattern of C_1_–C_3_ and C_2_–C_4_; therefore, this amino acid substitution likely disrupts the structure and function of CCP domain 7 and results in a free cysteine that may interact covalently with surrounding residues. Functional characterization of a different amino acid substitution at the same position of the peptide, p.(Cys431Tyr), showed that it decreased stability of the recombinant FH.^41^ Moreover, mass spectrometry analysis of plasma from the affected patient found that the protein product of the mutated allele was not present, suggesting that the change prevents secretion, the mutated protein is degraded rapidly, or both.^41^ The *CFH* p.(Arg127His) substitution alters CCP domain 2 in a region of FH and FHL-1 previously shown to be important for C3b binding and cofactor activity.^35^ In vivo investigations have shown that p.(Arg127His) results in retention of mutant full-length FH in the endoplasmic reticulum of cultured patient fibroblasts.^35^ Review of 29 *CFH* mutations previously reported as the underlying cause of EOMD (basal laminar drusen, cuticular drusen, and early-onset AMD) found that 75.8% impact FHL-1 as well as FH. This figure increases to 80.5% on inclusion of the variants reported by this study. Importantly, all 12 reported nonsense, splice-altering, and frameshift variants (5 by this study, 7 reported previously in the literature) fall within CCP domains 1 through 7. Furthermore, all missense substitutions (n = 10) affecting CCP domains 1 through 7 that have been evaluated in vitro have been found to prevent secretion or to result in loss of protein function. Consequently, at least 81% of reported EOMD variants result in loss of protein function, strongly supporting the hypothesis that haploinsufficiency of FHL-1 is one important mechanism underpinning the development of drusen in early adulthood. The contribution of AMD-related loci to EOMD, if any, remains elusive, but is an important area for future research. Moreover, identification of higher numbers of affected individuals or families likely would be required to associate demographics such as age at onset and severity with AMD risk alleles in this subgroup of patients.

Strict control of innate immunity at BrM is critical for maintaining normal homeostasis and health of the retina. It is becoming increasingly apparent that one of the main pathologic characteristics of AMD is inflammation of the central retina and consequential particulate accumulation (drusen development), cellular damage, and subsequent loss of vision as a result of complement dysregulation.^42^ Factor H, and almost certainly FHL-1, are the only components of the complement system known to downregulate alternative pathway activation on host extracellular matrix and self-surfaces via interaction with binding partners and cofactors.^12^, ^39^ There is increasing evidence that the eye synthesizes complement pathway components locally,^5^, ^43^, ^44^ and the importance of functional FHL-1 at the retina is becoming ever more apparent.^26^, ^27^, ^40^

It is known that FHL-1 is the only isoform that can diffuse passively across BrM; FH cannot because of its large size.^26^ Factor H-like 1 is immobilized in BrM and the ECM of the choriocapillaris by interaction with heparan sulfate via its glycosaminoglycan-binding site in CCP domain 7. In this way, FHL-1 functions to protect the ECM from complement activation.^26^ The Y402H polymorphism has been shown to affect the function of FHL-1; the 402H variant reduces FHL-1 binding to heparan sulphate.^26^ According to the GnomAD database, 44.08% of individuals from all populations (42.6% in the European [non-Finnish] population) are heterozygous for the 402H allele, whereas 32.54% of all populations (38.4% of the European [non-Finnish] population) are homozygous. Individuals heterozygous for the 402H variant are at a 2-fold increased risk of AMD, whereas those who are homozygous have a more than 5-fold increased risk.^4^ Studies have shown that although the AMD-associated Y402H allele does not alter FH protein conformation, nor does it alter FH levels in blood. It does result in decreased ability of FHL-1 to bind heparan sulfate^26^, ^45^, ^46^; any changes to FH binding to glycosaminoglycans seems minimal because of this larger protein’s second glycosaminoglycan-binding site. Furthermore, recent work has shown that the Y402H polymorphism has a more pronounced effect on FHL-1 binding of pentraxin 3—an inflammation-associated protein that binds FH at CCP domain 7 and CCP domains 19 and 20 and increases interaction of FH with apoptotic cells for iC3b opsonization—than it does on FH binding of pentraxin 3, most likely because it affects the only pentraxin 3 binding site within FHL-1.^40^ Taken together, this evidence suggests that loss of FHL-1 expression or function has a detrimental impact on regulation of the complement system in the retina.

Previous publications that have identified rare, highly penetrant *CFH* mutations in association with EOMD have focused on the location of mutations within the FH protein with respect to known functions of domains.^23^, ^37^ However, it is now recognized that FHL-1 is the predominant regulator of the complement pathway within BrM and the intercapillary septa.^26^ Factor H and FHL-1 both negatively regulate the alternative complement pathway by competing for binding to C3b with factor B to govern the removal of immune complexes and pathogens and to modulate adaptive immunity. They also serve as cofactors for factor I cleavage of C3b into its hemolytically inactive state, iC3b. We suggest that pathogenic variations in FHL-1 resulting in loss or impairment of function are an important cause of EOMD in the vast majority of patients.

There exists a well-recognized genotype–phenotype correlation with respect to *CFH* variants and disease, with variants affecting CCP domains 1 through 4 and CCP domains 6 through 8 mainly causing eye disease, whereas those affecting CCP domains 19 and 20 causing kidney disease.^17^ This is supported by research that has shown the glycosaminoglycan-binding site in CCP domain 7 demonstrates selectivity toward heparan sulfates in BrM and the choriocapillaris, whereas the region of CCP domains 19 and 20 preferentially binds heparan sulfates in the glomerular basement membrane of the kidneys.^12^, ^47^ However, a degree of allelic overlap exists that cannot be explained by our current understanding of the function of FH and FHL-1, representing an important area for future research.

In conclusion, rare, deleterious mutations in *CFH* resulting in haploinsufficiency of FH and FHL-1 are an important and underrecognized cause of dominant EOMD. Identification of *CFH* variants underlying EOMD has important consequences for clinical care, allowing genetic testing of other family members and counseling where appropriate; the identification of the underlying molecular cause can allow the provision of more accurate prognostic information, particularly where variants are known to increase the risk of progressing to a severe phenotype resulting in significant visual loss.^10^, ^48^ Furthermore, with complement-modulating therapeutics being developed for AMD, such genetic analyses may identify subsets of patients who may benefit from these new treatments. The impact of variants on the expression or function of FHL-1, or both, have not been considered previously for EOMD, and we propose that this truncated form of FH plays a crucial role in EOMD and potentially AMD. Identification of further mutations causing the rare, genetically heterogeneous EOMD phenotype will lead to a better understanding of disease pathogenesis.

## References

[1] Wong W.L., Su X., Li X. (2014). Global prevalence of age-related macular degeneration and disease burden projection for 2020 and 2040: a systematic review and meta-analysis. Lancet Glob Health.

[2] Liszewski M.K., Java A., Schramm E.C., Atkinson J.P. (2017). Complement dysregulation and disease: insights from contemporary genetics. Annu Rev Pathol.

[3] Schramm E.C., Clark S.J., Triebwasser M.P. (2014). Genetic variants in the complement system predisposing to age-related macular degeneration: a review. Mol Immunol.

[4] Fritsche L.G., Fariss R.N., Stambolian D. (2014). Age-related macular degeneration: genetics and biology coming together. Annu Rev Genomics Hum Genet.

[5] Hageman G.S., Anderson D.H., Johnson L.V. (2005). A common haplotype in the complement regulatory gene factor H (HF1/CFH) predisposes individuals to age-related macular degeneration. Proc Natl Acad Sci U S A.

[6] Edwards A.O., Ritter R., Abel K.J. (2005). Complement factor H polymorphism and age-related macular degeneration. Science.

[7] Maller J.B., Fagerness J.A., Reynolds R.C. (2007). Variation in complement factor 3 is associated with risk of age-related macular degeneration. Nat Genet.

[8] Fritsche L.G., Chen W., Schu M. (2013). Seven new loci associated with age-related macular degeneration. Nat Genet.

[9] Fritsche L.G., Igl W., Bailey J.N. (2016). A large genome-wide association study of age-related macular degeneration highlights contributions of rare and common variants. Nat Genet.

[10] Raychaudhuri S., Iartchouk O., Chin K. (2011). A rare penetrant mutation in CFH confers high risk of age-related macular degeneration. Nat Genet.

[11] Parente R., Clark S.J., Inforzato A., Day A.J. (2017). Complement factor H in host defense and immune evasion. Cell Mol Life Sci.

[12] Clark S.J., Bishop P.N. (2015). Role of factor H and related proteins in regulating complement activation in the macula, and relevance to age-related macular degeneration. J Clin Med.

[13] Thurman J.M., Holers V.M. (2006). The central role of the alternative complement pathway in human disease. J Immunol.

[14] Holers V.M. (2008). The spectrum of complement alternative pathway-mediated diseases. Immunol Rev.

[15] Hourcade D., Holers V.M., Atkinson J.P. (1989). The regulators of complement activation (RCA) gene cluster. Adv Immunol.

[16] Clark S.J., Ridge L.A., Herbert A.P. (2013). Tissue-specific host recognition by complement factor H is mediated by differential activities of its glycosaminoglycan-binding regions. J Immunol.

[17] Kavanagh D., Goodship T. (2010). Genetics and complement in atypical HUS. Pediatr Nephrol.

[18] Triebwasser M.P., Roberson E.D., Yu Y. (2015). Rare variants in the functional domains of complement factor H are associated with age-related macular degeneration. Invest Ophthalmol Vis Sci.

[19] Geerlings M.J., de Jong E.K., den Hollander A.I. (2017). The complement system in age-related macular degeneration: a review of rare genetic variants and implications for personalized treatment. Mol Immunol.

[20] Boon C.J., Klevering B.J., Hoyng C.B. (2008). Basal laminar drusen caused by compound heterozygous variants in the CFH gene. Am J Hum Genet.

[21] van de Ven J.P., Boon C.J., Fauser S. (2012). Clinical evaluation of 3 families with basal laminar drusen caused by novel mutations in the complement factor H gene. Arch Ophthalmol.

[22] Yu Y., Triebwasser M.P., Wong E.K. (2014). Whole-exome sequencing identifies rare, functional CFH variants in families with macular degeneration. Hum Mol Genet.

[23] Duvvari M.R., Saksens N.T., van de Ven J.P. (2015). Analysis of rare variants in the CFH gene in patients with the cuticular drusen subtype of age-related macular degeneration. Mol Vis.

[24] Hughes A.E., Meng W., Bridgett S., Bradley D.T. (2016). Rare CFH mutations and early-onset age-related macular degeneration. Acta Ophthalmol.

[25] Wagner E.K., Raychaudhuri S., Villalonga M.B. (2016). Mapping rare, deleterious mutations in factor H: association with early onset, drusen burden, and lower antigenic levels in familial AMD. Sci Rep.

[26] Clark S.J., Schmidt C.Q., White A.M. (2014). Identification of factor H-like protein 1 as the predominant complement regulator in Bruch’s membrane: implications for age-related macular degeneration. J Immunol.

[27] Clark S.J., McHarg S., Tilakaratna V. (2017). Bruch’s membrane compartmentalizes complement regulation in the eye with implications for therapeutic design in age-related macular degeneration. Front Immunol.

[28] Taylor R.L., Arno G., Poulter J.A. (2017). Association of steroid 5alpha-reductase type 3 congenital disorder of glycosylation with early-onset retinal dystrophy. JAMA Ophthalmol.

[29] Falcao D.A., Reis E.S., Paixao-Cavalcante D. (2008). Deficiency of the human complement regulatory protein factor H associated with low levels of component C9. Scand J Immunol.

[30] Servais A., Noel L.H., Roumenina L.T. (2012). Acquired and genetic complement abnormalities play a critical role in dense deposit disease and other C3 glomerulopathies. Kidney Int.

[31] Dragon-Durey M.A., Fremeaux-Bacchi V., Loirat C. (2004). Heterozygous and homozygous factor h deficiencies associated with hemolytic uremic syndrome or membranoproliferative glomerulonephritis: report and genetic analysis of 16 cases. J Am Soc Nephrol.

[32] Schmidt B.Z., Fowler N.L., Hidvegi T. (1999). Disruption of disulfide bonds is responsible for impaired secretion in human complement factor H deficiency. J Biol Chem.

[33] Merinero H.M., Garcia S.P., Garcia-Fernandez J. (2018). Complete functional characterization of disease-associated genetic variants in the complement factor H gene. Kidney Int.

[34] Pechtl I.C., Kavanagh D., McIntosh N. (2011). Disease-associated N-terminal complement factor H mutations perturb cofactor and decay-accelerating activities. J Biol Chem.

[35] Albuquerque J.A., Lamers M.L., Castiblanco-Valencia M.M. (2012). Chemical chaperones curcumin and 4-phenylbutyric acid improve secretion of mutant factor H R127H by fibroblasts from a factor H-deficient patient. J Immunol.

[36] Ferreira V.P., Herbert A.P., Cortes C. (2009). The binding of factor H to a complex of physiological polyanions and C3b on cells is impaired in atypical hemolytic uremic syndrome. J Immunol.

[37] Duvvari M.R., van de Ven J.P., Geerlings M.J. (2016). Whole exome sequencing in patients with the cuticular drusen subtype of age-related macular degeneration. PLoS One.

[38] Schejbel L., Schmidt I.M., Kirchhoff M. (2011). Complement factor H deficiency and endocapillary glomerulonephritis due to paternal isodisomy and a novel factor H mutation. Genes Immun.

[39] Clark S.J., Bishop P.N. (2018). The eye as a complement dysregulation hotspot. Semin Immunopathol.

[40] Swinkels M., Zhang J.H., Tilakaratna V. (2018). C-reactive protein and pentraxin-3 binding of factor H-like protein 1 differs from complement factor H: implications for retinal inflammation. Sci Rep.

[41] Montes T., Goicoechea de Jorge E., Ramos R. (2008). Genetic deficiency of complement factor H in a patient with age-related macular degeneration and membranoproliferative glomerulonephritis. Mol Immunol.

[42] McHarg S., Clark S.J., Day A.J., Bishop P.N. (2015). Age-related macular degeneration and the role of the complement system. Mol Immunol.

[43] Mandal M.N., Ayyagari R. (2006). Complement factor H: spatial and temporal expression and localization in the eye. Invest Ophthalmol Vis Sci.

[44] Hallam D., Collin J., Bojic S. (2017). An induced pluripotent stem cell patient specific model of complement factor H (Y402H) polymorphism displays characteristic features of age-related macular degeneration and indicates a beneficial role for UV light exposure. Stem Cells.

[45] Clark S.J., Higman V.A., Mulloy B. (2006). His-384 allotypic variant of factor H associated with age-related macular degeneration has different heparin binding properties from the non-disease-associated form. J Biol Chem.

[46] Clark S.J., Perveen R., Hakobyan S. (2010). Impaired binding of the age-related macular degeneration-associated complement factor H 402H allotype to Bruch’s membrane in human retina. J Biol Chem.

[47] Saunders R.E., Abarrategui-Garrido C., Fremeaux-Bacchi V. (2007). The interactive factor H-atypical hemolytic uremic syndrome mutation database and website: update and integration of membrane cofactor protein and factor I mutations with structural models. Hum Mutat.

[48] Ferrara D., Seddon J.M. (2015). Phenotypic characterization of complement factor H R1210C rare genetic variant in age-related macular degeneration. JAMA Ophthalmol.

