# Supplementary material for: Loss-of-Function Mutations in the CFH Gene Affecting Alternatively Encoded Factor H-like 1 Protein Cause Dominant Early-Onset Macular Drusen
Source: Ophthalmology. 2019 Oct;126(10):1410–21. doi: 10.1016/j.ophtha.2019.03.013 (PMC6856713; doi:10.1016/j.ophtha.2019.03.013)
Supplement: Figure S1 [file mmc1.pdf]

Figure 1

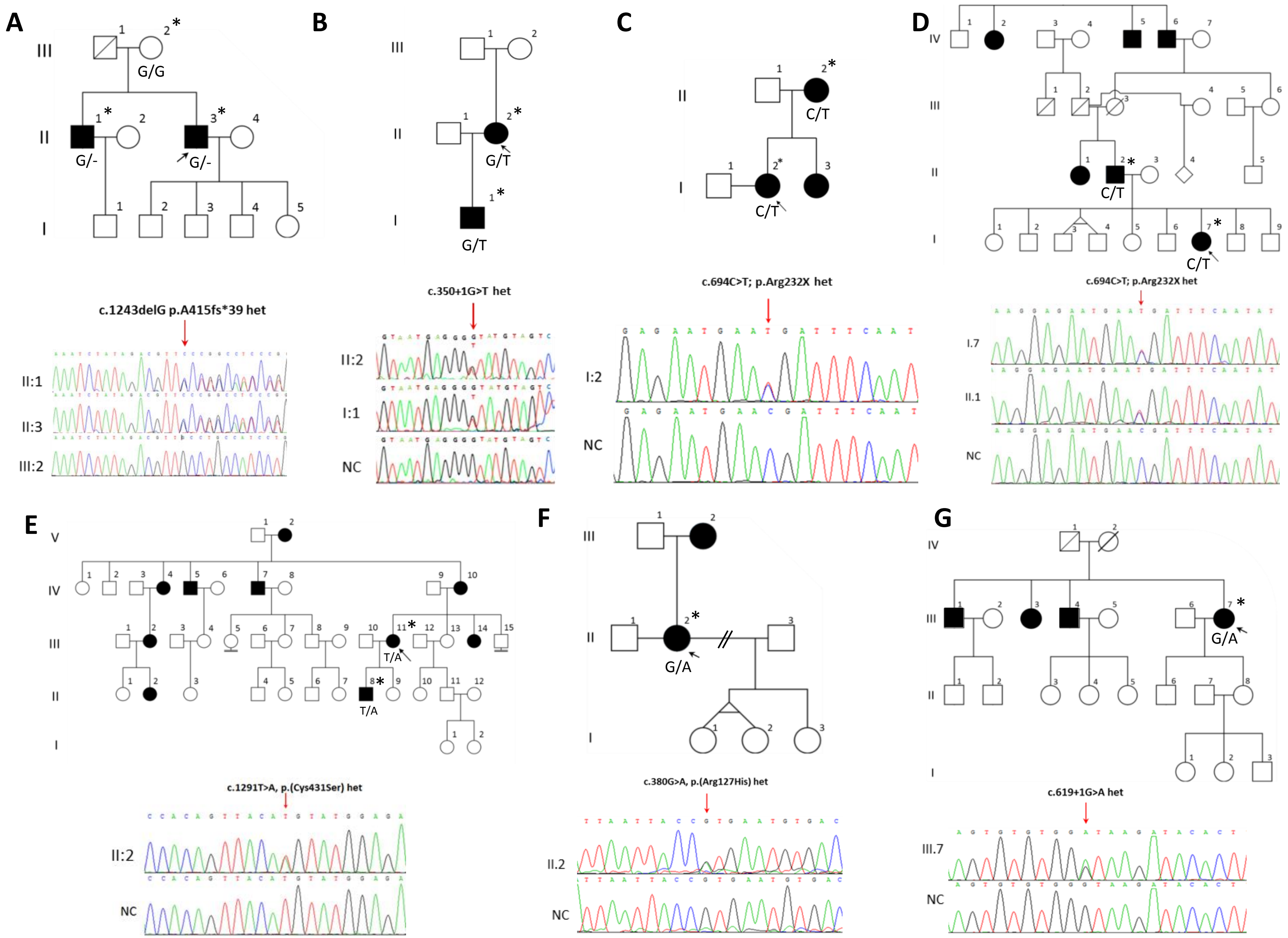

Pedigrees and Sanger sequencing chromatograms of identified CFH variants (Families A-G): Pedigrees detailing family history of early onset macular generation in each case alongside corresponding sequencing chromatogram indicating the identified *CFH* mutation (indicated by red arrow). Letters indicate family identifier; black squares/circles indicate affected family members; white squares/circles indicate unaffected family members; asterisks (*) indicate individuals with DNA available and in whom the CFH mutation was confirmed as present. Genetic status is indicated underneath the symbol for each individual tested.
