# Supplementary material for: Loss-of-Function Mutations in the CFH Gene Affecting Alternatively Encoded Factor H-like 1 Protein Cause Dominant Early-Onset Macular Drusen
Source: Ophthalmology. 2019 Oct;126(10):1410–21. doi: 10.1016/j.ophtha.2019.03.013 (PMC6856713; doi:10.1016/j.ophtha.2019.03.013)
Supplement: Figure S2 [file mmc2.pdf]

Figure 2

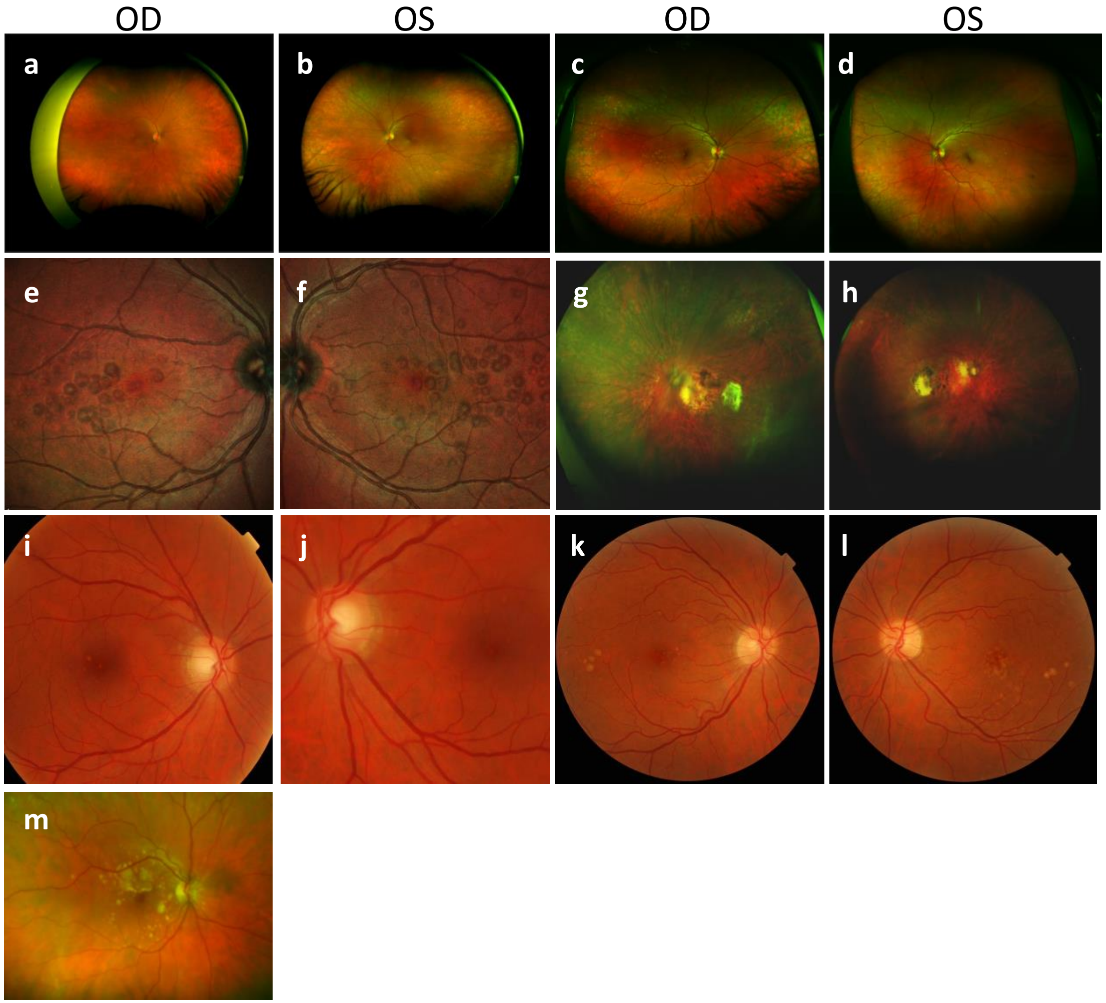

Colour fundus images in patients with CFH mutations (a-m). a-b) Patient A:II.3 aged 51 years: Wide-fields colour fundus photographs showing wide-spread drusen and mottling of the RPE at the fovea; **c-d)** Patient A:II.1 aged 49 years: Wide-field colour fundus imaging found bilateral widespread drusen; **e-f)** Patient D:I.7 aged 26 years: Wide-field colour fundus images showing multiple colloid drusen within and surrounding the macular, bilaterally; **g-h)** Patient E:II.2 aged 52 years: Colour fundus imaging showing bilateral macular atrophic changes with temporal retinal pigmentary and fibrotic changes, and peripheral drusen; **i-j)** Patient E:III.2 aged 40 years: Colour fundus imaging revealed small, sparse drusen at the maculae; **k-l)** Patient F:II.2 aged 54: colour fundus photos showing retinal drusen at the fovea within the arcades, and temporal to the macula; **m)** Patient G:III.7 aged 66: Multicolour fundus image of right eye showing soft confluent drusen with focal areas of retinal pigment hyperplasia at posterior pole and nasal to disc. Images a-h and m are from an Optos Confocal Scanning Laser Ophthalmoscope and thus do not represent real colour.
