# Supplementary material for: Loss-of-Function Mutations in the CFH Gene Affecting Alternatively Encoded Factor H-like 1 Protein Cause Dominant Early-Onset Macular Drusen
Source: Ophthalmology. 2019 Oct;126(10):1410–21. doi: 10.1016/j.ophtha.2019.03.013 (PMC6856713; doi:10.1016/j.ophtha.2019.03.013)
Supplement: Figure S4 [file mmc3.pdf]

Figure 4

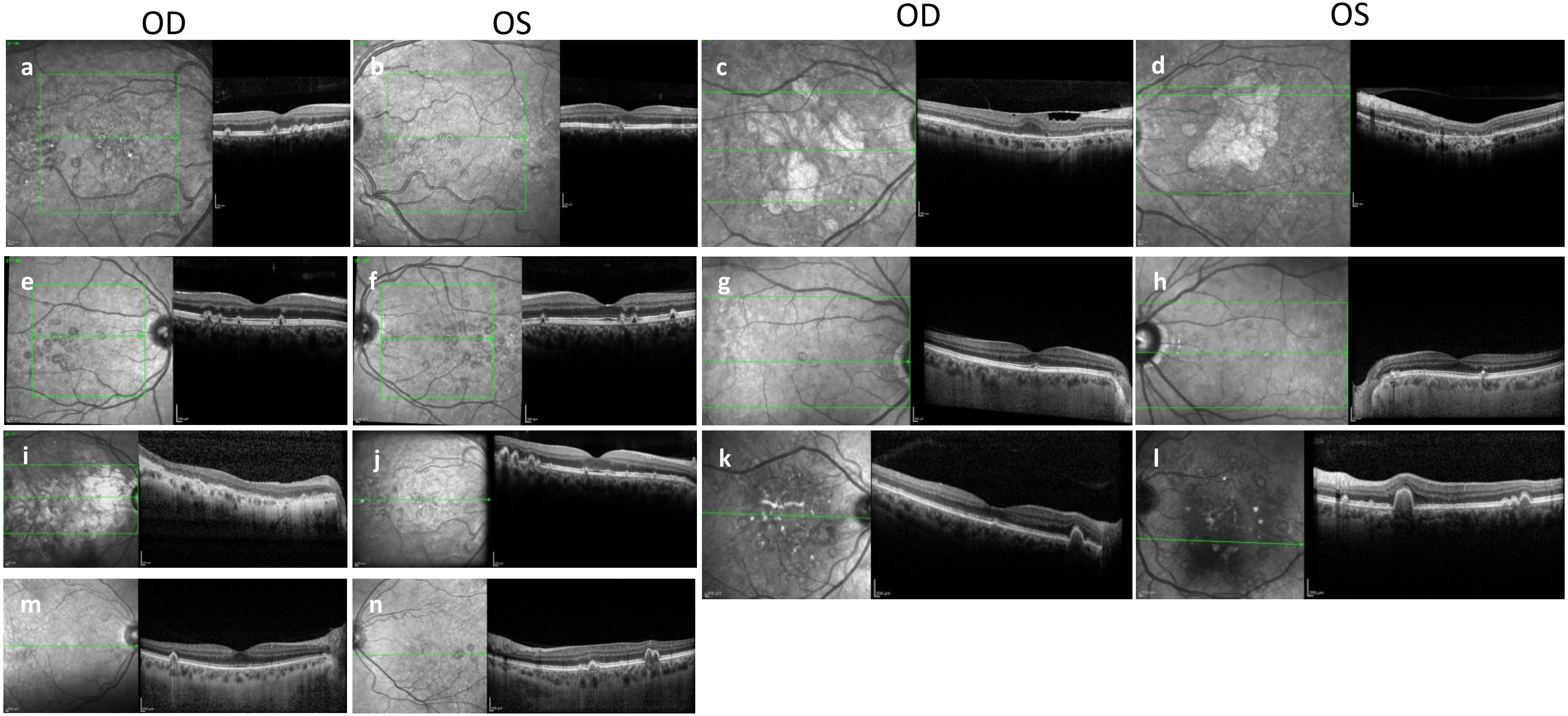

Optical coherence tomography scans (OCTs) in patients with CFH mutations (a-n). OCTs are shown as vertical scans, at 30 degrees through the fovea. **a-b)** Patient A:II.3 aged 51 years: OCT scan confirming the presence of small dome shaped elevations within the retinal pigment epithelium (RPE)/Bruch's membrane; **c-d)** Patient C:I.2 aged 64 years: OCT scan showing central atrophy with epiretinal changes; **e-f)** Patient D:I.7 aged 26 years: OCT scan showing multiple small dome shaped elevations affecting the RPE/Bruch's membrane that correspond with the location of the large colloid drusen within the macular in the en face infra red image; **g-h)** Patient E:III.2 aged 52 years: OCT through fovea showing few large drusen at the maculae; **i)** Patient E:II.2 aged 53 years: En face infra-red image of the macular showing widespread atrophy and accompanying OCT showing loss of the ellipsoid layer and RPE loss; **j)** Patient A:II.1 aged 49 years: En face infra-red imaging showed drusen were also present within the macular of the RE and concentrated in an area temporal to the macular, bilaterally, that appeared denser in the RE. OCT showed multiple RPE elevations that disrupted the outer photoreceptor layer and extended in to the mid-periphery; **k-l)** Patient G:III.7 aged 66 years: OCT showing abrupt drusenoid pigment epithelial detachments and drusen; **m-n)** Patient F:II.2 aged 54: En face images showing temporal drusen demonstrated an increased signal on infa-red signal. OCTs show steep sided drusen in the temporal regions of both eyes with smaller drusen at the maculae.
