# Supplementary material for: Loss-of-Function Mutations in the CFH Gene Affecting Alternatively Encoded Factor H-like 1 Protein Cause Dominant Early-Onset Macular Drusen
Source: Ophthalmology. 2019 Oct;126(10):1410–21. doi: 10.1016/j.ophtha.2019.03.013 (PMC6856713; doi:10.1016/j.ophtha.2019.03.013)
Supplement: Supplemental Data A [file mmc4.pdf]

Supplementary Data A: Supplementary methods

Whole exome sequencing

Genomic DNA was extracted from peripheral blood samples according to local protocols. DNA libraries for WES were prepared using 200ng of high quality genomic DNA. Exons and 50bp of flanking intronic sequence were captured using the SureSelect Human All Exon V6 enrichment kit (Agilent, Santa Clara, Ca, USA) or SureSelect Focussed Exome kit (Agilent) (Family B)and sequenced on the HiSeq2500 (Illumina, Inc. San Diego, Ca, USA). Sequencing reads were aligned to the GRCh37/Hg19 human reference genome using Novoalign v2.08.03 (Novocraft Technologies Sdn Bhd, Selangor, Malaysia) or bwa (v0.7.12), and processed for duplicates and in/del realignment using Picard or a combination of Samtools, Picard and the Genome Analysis Toolkit (GATK) (family B), according to best practice guidelines. Variant calling was conducted using the Genome Analysis Tool Kit (GATK, version 3.3-0) and annotated using ANNOVAR, based on Ensembl v75 gene and transcript definitions. For Family B, variant calling was performed using the HaplotypeCaller function of GATK. Variants with a minor allele frequency ≥ 1% in dbSNP142 and ExAC were removed. Information on variant frequency was generated with an internal database of approximately 4000 exomes (UCLex dataset), and external datasets from NHLBI GO Exome Sequencing Project (ESP), Exome Aggregation Consortium (ExAC), and gnomAD database. Additional annotation was provided from PhyloP, SIFT, PolyPhen2, Mutation Taster, GERP and OMIM (http://omim.org/), to assist analysis and determination of variant pathogenicity. For family B, variants were annotated using Variant Effect Predictor, CADD scores calculated.

Sanger Sequencing

The coding exons and flanking intronic sequences of *CFH* (NM_000186) plus an additional four amino acids unique to the alternative transcript, FHL-1 (exon 10, NM_0010149975) were amplified by PCR and subject to bi-directional Sanger sequencing (see supplementary data A for details). Primers were designed using Primer3Plus software (http://primer3plus.com/) and N13-tags were

added to each oligo nucleotide to facilitate sequencing. Sequencing was performed using BigDye Terminator v3.1 Cycle Sequencing Kit (Applied Biosystems Corporation, Ca, USA) and products analysed using the 3730XL (Applied Biosystems, Waltham, CA, USA) according to manufacturers' instructions . Variants identified by WES were also validated by bi-directional Sanger sequencing.

Plasmid Preparation and Validation

The *CFH* cDNA sequence for transcript NM_001014975 that encodes human FHL-1, flanked by a 5' HindIII site followed by a Kozak sequence (5'-GCCACC-3') and 3' EcoRV site, and with an additional sequence coding for six histidine residues (5'-CACCACCACCATCACCAC-3') inserted downstream of the signal peptide (amino acids 1-18) to create an N-terminal His-tag, was synthesised, mutated by site-directed mutagenesis (to generate FHL-1_{R127H} and FHL-1_{C431S}) and directionally cloned in to pcDNA3.1 by GeneArt Gene Synthesis (Thermofisher Scientific, MA,USA). The *CFH* c.1243del, p.(Ala415ProfsTer47) mutation identified in Family A was predicted to result in a frameshift starting in the penultimate exon (exon 9) of NM_001014975. Thus, a mutant sequence (FHL-$1_{A415Pf/s}$) with restrictions sites, Kozac sequence and N-terminal His-tag (as above) was designed to include 47 nucleotides of the 3'UTR that were predicted to be incorporated in to the mutated protein sequence as a result of the shift in reading-frame, for synthesis and directional cloning in to pcDNA3.1 (as above) by GeneArt Gene Synthesis (Thermofisher Scientific). A common polymorphism, c.1204C>T; p.(Tyr402His) (MAF:0.67), exists within NM_001014975. The His402 variant is common in the general population (MAF: 44.08%) has been shown to impair FHL-1 function[26], thus all constructs were generated to encode the reference (Hg19) tyrosine at this position (402Y). Each construct was sequence verified by Sanger sequencing and found to be 100% congruent..

FHL-1 is a cofactor for the serine-protease factor I-mediated proteolysis of C3b which generates iC3b in the form of an unaltered β-chain along with two α-chain cleavage products of 68 and 43kDa. Thus, to determine whether the His-tag of the FHL-1 construct affected the normal function of the protein we measured its ability to act as a co-factor for factor I-catalysed cleavage of C3b to iC3b by

comparison with un-tagged FHL-1. For this, a gradient of concentrations of His-tagged or untagged-FHL-1 was incubated with C3b and factor I in PBS prior to analysis by SDS-PAGE. Our data show that iC3b production, as indicated by the disappearance of the C3b α-chain band and appearance of the two subsequent breakdown products at 68 and 43kDa, was comparable between His-tagged and un-tagged FHL-1 (Figure A1). This data indicates that His-tagged FHL-1 interacts with C3b and facilitates productive interactions between C3b and factor I; thus providing evidence that the N-terminal His-tag does not affect the function of the recombinant protein.

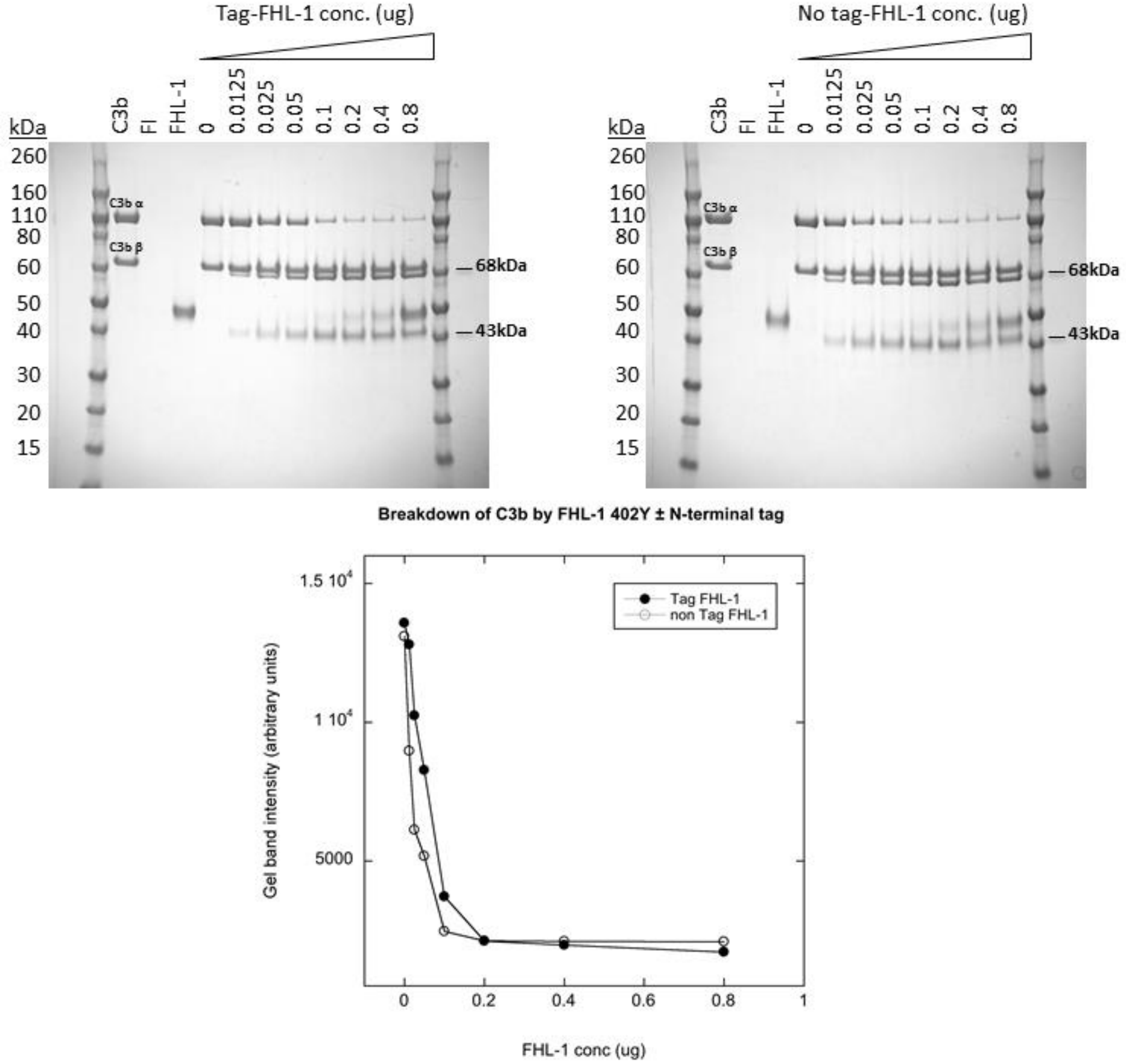

Supplementary Figure A1: Cofactor assay measuring C3b breakdown by recombinant FHL-1 ± N-terminal His-tag. The abilities of (A) His-tagged FHL-1 ('Tag-FHL-1'; lanes 4-11) versus (B) untagged FHL-1 ('no tag-FHL-1'; lanes 4-11), to act as co-factors for factor I-catalysed cleavage of C3b to iC3b were assessed by visualising the 43-kDa and 68-kDa proteolytic fragments of the α'-chain using SDS–PAGE followed by Coomassie blue staining. FHL-1 concentrations were 0-0.8ug, as indicated. (C) The band intensity of the C3b α-chain is plotted for both tagged and non-tagged FHL-1 for comparison.

Plasmid Transformation

Plasmids were transformed by adding 200ng of plasmid to 100uL of MAX Efficiency DH5α™-T1 chemically competent bacterial cells (Life Technologies, CA, USA). Briefly, DNA was added to the cells and incubated on ice for 30mins. Cells were heat shocked in a water bath at 42°C for 1 minute then cooled on ice for 2 minutes. S.O.C media (2% (w/v) tryptone, 0.5% (w/v) yeast extract, 20mM glucose, 10mM NaCl, 2.5mM KCl, 5mM $MgCl_2$, 5mM MgSO4) (Life Technologies) was added to the cell suspension and incubated at 37°C for 1 hour with shaking at 220rpm. Selection of clones containing the expression plasmid was achieved by spreading the transformed bacterial cells on to LB agar plates containing 50mg/ml ampicillin. Plates were incubated at 37°C for 16 hours. Resulting colonies were considered likely to carry multiple copies of the target genes. Colonies were picked and grown in LB broth containing 200µg ml^{-1} ampicillin at 37°C shaking (220rpm) for 16 hours. Cultured bacterial cells were pelleted by centrifugation and plasmid DNA was purified using the QIAGEN Endofree Plasmid MegaKit (QIAGEN, Hilden, Germany), according to manufacturer's instructions.

TransientTransfection of FHL-1 Plasmids and Purification of Recombinant Proteins

Twenty-four hours prior to transfection, $7X10^6$ cells were seeded in 20cm petri dishes. Purified plasmids were stably transfected in to HEK293 cells using polyethylenimine (PEI). Transfected cells were incubated for 5 hours at 37°C with 5% CO_2 before replacement of the transfection media with Dulbecco's Modified Eagle's Medium (D6429, Sigma) with 2% heat-inactivated fetal bovine serum (FBS) (Life Technologies). Media was harvested from transfected cells at 24, 48, 72, and 144 hours. Phenylmethylsulfonyl fluoride (6.25mM v/v final) was added to inhibit protease activity before collected media was centrifuged at 4000rpm for 15min to collect cellular debris. Harvested media was pooled prior to purification of recombinant proteins.

The collected media was buffered to pH7.5 prior to the addition of Amintra Ni-NTA affinity resin (Expedeon, CA, USA) and rolling incubation overnight at 4°C. Beads were isolated from the media using a PD-10 column (Merck-Millipore, MA, USA) and washed with 50 mM HEPES, 500 mM NaCl, 20 mM Imidazole, pH 7.5 by gravity flow. Recombinant proteins were eluted from the beads using 50 mM HEPES, 500 mM NaCl, 500 mM Imidazole, pH 7.5 and dialysed with 20 mM HEPES, 250 mM NaCl, pH 9.0. Concentrations of secreted proteins were determined by measuring the OD280/extinction co-efficient. Protein purity was determined by running 20uL of purified protein eluate on a 4-12% SDS gel before staining for total protein using coomassie blue. Figure A2 depicts the contents of various fractions throughout the purification procedure, demonstrating the successful isolation of purified His-tagged FHL-1 from the media collected from transfected cells.

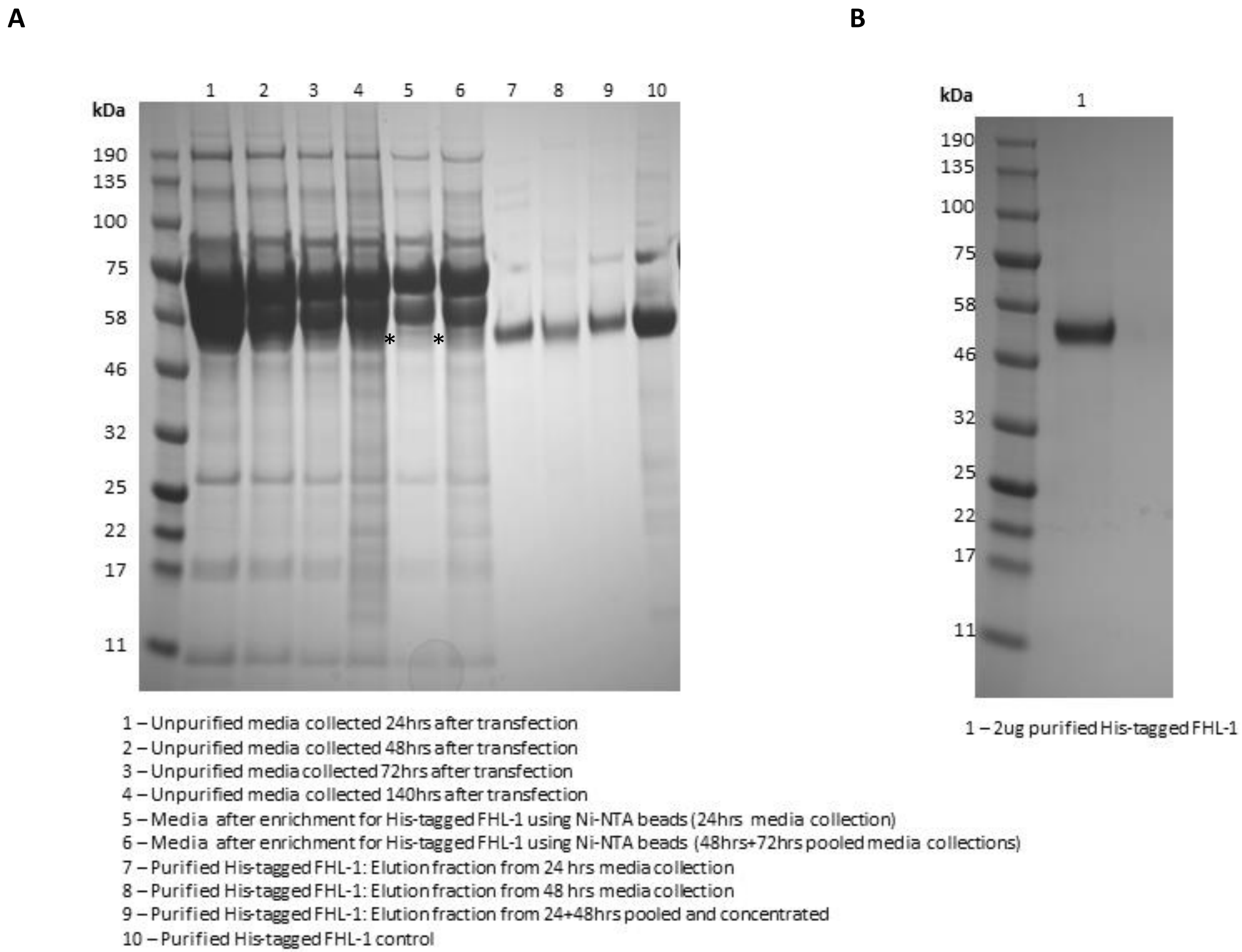

Figure A2: Purification of His-tagged FHL-1 from media collections. Fractions of media collections were analysed by SDS-PAGE and visualised using coomassie blue staining. (A) Lanes 1-4 show contents of unpurified media collected a 24-140 hours following transfection; lanes 5-6 show contents of collected media fractions following enrichment and isolation of His-Tagged FHL-1 using Ni-NTA beads (*note absence of ~51kDa product in these lanes which represents the successful isolation of the His-tagged recombinant protein);lanes 7-9 show purified His-tagged FHL-1 in the elution fractions of medais harvested at 24 hours, 48 hours and 24-48 hour pooled sample; lane 10 shows purified His-tagged FHL-1 from a previous purification, used as a size control. (B) 2ug of purified His-tagged FHL-1.

Preparation of Cell Lysates

HEK293T cells were detached using 0.25% trypsin (Thermo Fisher Scientific) and pelleted by centrifugation. Cell pellets were washed in PBS (-$Mg2^{+}$/-$Ca2^{+}$) and lysed in RIPA buffer (Sigma-Aldrich, MO, USA) containing cOmplete™, EDTA-free Protease Inhibitor Cocktail (Sigma-Aldrich), and incubated on ice for 30 minutes. Lysates were vortexed briefly, centrifuged to pellet debris and the supernatant retained. Lysates were quantified using the Pierce BCA protein assay according to manufaturer's protocols.

Western Blotting

Equal amounts of purified recombinant proteins and 50µg cell lysates were electrophoresed on 4–12% NuPAGE Bis-Tris gels (Life Technologies) at 200 V for 60 min and transferred onto nitrocellulose membranes at 80 mA for 1.5 h using semidry transfer apparatus in transfer buffer (25 mM Tris, 192 mM glycine, 10% [v/v] methanol). The membranes were blocked in PBS, 10% (w/v) milk, and 0.2% (w/v) BSA for 16 h at 4°C before the addition of murine monoclonal Anti-6X His tag (Abcam) for detection of the N-terminal His-tag of recombinant proteins, and rabbit polyclonal SOD2 antibody (Sigma-Aldrich) as a loading control for cell lysates, in PBS with 0.2% (v/v) Tween 20 (PBS-T) for 2 h at room temperature. Membranes were washed twice for 30 min in 0.2% PBS-T before the addition of Anti-mouse (6X His) or anti-rabbit (SOD2) IgG, HRP-conjugated antibody (Cell Signaling Technology, Herts, UK) at 1:2500 dilution in 0.2% PBS-T, and washed again as before. Membranes were developed using SuperSignal West Pico PLUS Chemiluminescent Substrate (Thermo Fisher Scientific) for 3 min at room temperature, according to manufacturer's instructions. Reactive bands were detected by exposing Fuji RX-Ray Film to the treated membrane for 10 minutes at room temperature and developed on an automated film developer.
