# Supplementary material for: Loss-of-Function Mutations in the CFH Gene Affecting Alternatively Encoded Factor H-like 1 Protein Cause Dominant Early-Onset Macular Drusen
Source: Ophthalmology. 2019 Oct;126(10):1410–21. doi: 10.1016/j.ophtha.2019.03.013 (PMC6856713; doi:10.1016/j.ophtha.2019.03.013)
Supplement: Supplemental Data B [file mmc5.pdf]

Supplementary Data B: Detailed Ophthalmic Histories and Retinal Phenotypes

Family A Patient A:I.1 was found to harbour minor, visually insignificant retinal changes when assessed by an optometrist at 18 years of age that were not investigated any further. Additional changes were observed at 41 years which prompted referral to an ophthalmologist. Visual assessment found mild hyperopia (Table 1). Ophthalmic assessment at 47 years identified 'globular' drusen and the patient described his vision appearing darker in his right eye (OD) (Figure 3). Multi-colour fundus imaging of the right macular showed drusen scattered throughout with mottling of the retinal pigment epithelium (RPE) at the fovea (Figure 2). FAF showed multiple drusen scattered throughout the macular with irregular patches of hyperautofluorescent stippling. OCT confirmed the presence of small dome shaped elevations within the retinal pigment epithelium (RPE) (Figure 4). Nasal to the fovea, OCT indicated disruption of the photoreceptor and RPE interdigitation layers. Electroretinogram (ERG) was normal and grossly symmetrical in both light- and dark-adapted states. Patient A:I.3, the elder brother of I.1, began to experience visual problems in childhood and wore glasses from the age of 16 years. A routine optometry assessment at 42 years of age found good visual acuity (VA) in the right (OD) and left eye (OS), despite significant macular changes (Table 1 and Figure 3). The patient reported a decline in VA at 43-44 years when he also began to experience nyctalopia and photophobia. Visual assessment between the ages of 45-47 years found increasing hyperopia that coincided with the patient describing increasing difficulty completing detailed work, especially in dim light. Wide-field colour fundus imaging found bilateral widespread drusen and RPE changes in the peripheral retina that appeared more severe in the RE and was predominantly infero-nasal in the LE (Figure 2). Infra-red imaging showed drusen were also present within the macular of the OD and concentrated in an area temporal to the macular, bilaterally, that appeared denser in the OD. OCT showed multiple RPE elevations that disrupted the outer photoreceptor layer and extended in to the mid-periphery (Figure 4). ERG at 47 years found an asymmetrical rod cell abnormality restricted to the RE.

Family B The proband from family 2 (B:II.2) first noticed a loss in visual acuity towards the end of her eighth decade of life. Visual loss slowly progressed until her most recent exam aged 89 years when she was registered severely sight impaired. There were no major symptoms of nyctalopia. She had previously received treatment for cataract and bilateral dry macular degeneration in her OD. Retinal examination and imaging were consistent with bifocal outer retinal atrophy in a symmetrical pattern, bilaterally with sparing at the vertical meridian (Figure 3). She reported that her mother had poor vision and cataracts, however no details were available. Patient B:I.1, the affected son of B:II.2, underwent retinal examination at 61 years of age. Fundus visualisation revealed multiple drusen bilaterally that were present in a similar pattern to that of his affected mother, although less severe (Figure 3).

Family C Patient C:I.2, a 64 year old white female, was diagnosed with retinal drusen by her optometrist at the age of 54. Ophthalmic exam aged 64 years found central scotoma, and slow dark adaptation with photosensitivity. Visual acuities were 6/6 right and 6/15 left. Fundoscopy and colour imaging showed drusen surrounding the central atrophy extending into the arcades. There was also patchy atrophy in the peripheral retina with reticular and drusenoid features with mid peripheral sparing. Fundus autofluorescence showed loss of central signal consistent with atrophy, as well as drusen surrounding the atrophy and the optic nerve (Figure 3). OCT confirmed central

atrophy and epiretinal changes (Figure 4). Family history noted that her mother had received a clinical diagnosis of Tays honeycombe choroiditis' some years earlier, and her sister had also been diagnosed with macular degeneration.

Family D Patient D:II.2, a white male, presented at age 50 years with blurred vision. Ophthalmic examination found a right macular haemorrhage and drusen bilaterally. His visual acuities aged 64 years were 1/60 OD and 6/24 OS. He was diagnosed with macular retinal drusenoid dystrophy complicated by choroidal neovascularisation and significant scarring. Family history revealed that his maternal grandfather developed poor visual acuity in his forties, his maternal great aunt developed poor vision in her sixties. His mother died aged 56 years and it is not known if she was affected. His daughter, D:I.7, noticed changes in her vision at 26 years of age and ophthalmic examination revealed bilateral, large colloidal drusen at the maculae (Figure 3, Figure 2, Figure 4). At her most recent exam, aged 50 years, her visual acuities were 6/5 (OE).

Family E E:II.2 began to experience problems with her central vision, alongside photosensitivity and loss of colour vision at around 50 years of age. Family pedigree revealed a dominant history of early-onset macular drusen, with the probands mother, sibling, maternal aunt and cousin all similarly affected. Ophthalmic exam at 68 years of age found visual acuities of 6/120 (logMAR 1.34) OD and 6/96 OS. Ishihara scores were OD: 1/17 and OS: 2/17. Electrodiagnostic testing showed extinguished pattern ERG; whereas EOG revealed normal RPE function and ERG indicated photoreceptor function within normal limits. Fundus autofluorescence showed hypo-autofluorescence centrally with atrophy, and macula hyper-autofluorescence with a ring of hypo-autofluorescence surrounding the fovea, bilaterally (Figure 3). Fundus colour imaging showed bilateral macular atrophic changes with temporal retinal pigmentary and fibrotic changes, and peripheral drusen (Figure 2). Patient E:III.2, son of E.II.2, was found to have bilateral retinal drusen by his optometrist at age 40 years. He was asymptomatic with visual acuities of 6/5 OD and 6/6 OS. Fundoscopy revealed sparse small drusen Figure 2). Aged 52 years, he remained asymptomatic 6/4 OD and 6/7.5 OS. OCT found small sparse drusen at the macular, bilaterally, at 53 years of age (Figure 4).

Family F An asymptomatic white Caucasian female, presented at age 46 years of age for review as her Optometrist noted that she had macular drusen and her mother was being treated for neovascular age related macular degeneration. Her general medical history included taking methotrexate and folic acid for rheumatoid arthritis and thyroxine for hypothyroidism. Blood pressure was 120/80 and urinanalysis was normal. Fundoscopy revealed isolated sparse drusen within the macula and temporal raphes (Figure 2). The temporal drusen demonstrated an increased signal on autofluorescence imaging and optical coherence tomography showed minimal disruption of the retinal pigment epithelial layer with features most consistent with cuticular drusen (Figure 4). Visual acuities were 0.0 (Snellen equivalent 6/6) right and -0.1 left (6/4.8) left with -4/-1.00 x 180, and -4.50/-1.00 x 170. There were no new changes at her last review aged 54 years.

Family G An asymptomatic white Caucasian female, presented at age 45 years of age for review as her sister had been diagnosed with macular degeneration; her mother died age 30 and father died at 80 with no visual problems. She had no systemic disorders and blood pressure was 122/70 and urinanalysis was normal. Fundoscopy revealed large sparse white/yellow drusen at the maculae, nasal to the disc and surrounding the arcades (Figure 2). Most of the drusen demonstrated

an increased signal on autofluorescence imaging and there was some early non -foveal involving patchy geographic atrophy in the left eye. Optical coherence tomography showed increasing elevation of the retinal pigment epithelial layer with drusenoid pigment epithelial detachments at her last review (Figure 4). Visual acuities were 6/6 right and left with +2.75/-1.00 x 17, and +2.75/-1.50 x 165. She re-presented aged 53 with distortion, and a gradual increase in difficulties in reading. At her last review aged 66 years the visual acuities were unchanged; there were more drusen, but she was normotensive, and there was no evidence of geographic atrophy or choroidal neovascularisation, and she was discharged from follow up.
