# Supplementary material for: Loss-of-Function Mutations in the CFH Gene Affecting Alternatively Encoded Factor H-like 1 Protein Cause Dominant Early-Onset Macular Drusen
Source: Ophthalmology. 2019 Oct;126(10):1410–21. doi: 10.1016/j.ophtha.2019.03.013 (PMC6856713; doi:10.1016/j.ophtha.2019.03.013)
Supplement: Supplemental Data C [file mmc6.pdf]

Supplementary Data C

<table>
<tr><td colspan="2">A:II.3 and A:II.1 WES data filtering</td><td></td><td></td><td></td></tr>
<tr><td>Filter</td><td>Number of Variants</td><td></td><td></td><td></td></tr>
<tr><td>Rare Shared</td><td>301</td><td></td><td></td><td></td></tr>
<tr><td>Rare shared PAV</td><td>204</td><td></td><td></td><td></td></tr>
<tr><td rowspan="5">Rare shared PAV, known IRD genes</td><td rowspan="5">7</td><td>Variants of Interest</td><td>Interpretation</td><td>Justification</td></tr>
<tr><td>NM_000186.3(CFH):c.1243del, p.(Ala415ProfsTer39) het</td><td>Likely pathogenic mutation</td><td>Not present on gnomAD
Null variant in gene where LOF is a known mechanism of disease
Not previously reported as disease-causing</td></tr>
<tr><td>ENST00000264448.6(ALMS1):c.2033A>G, p.(Tyr678Cys)</td><td>Carrier of VUS</td><td>High allele frequency in European (non-Finnish) population (293/125986)
Heterozygous change in recessive gene
Gene does not correlate with patient phenotype
Predicted deleterious in silico (SIFT: Deleterious (0); Polyphen22 (probably damaging (0.997))
Not previously reported as disease-causing</td></tr>
<tr><td>NM_178857.5(RP1L1):c.6992C>T, p.(Thr2331Met) het</td><td>Benign</td><td>High allele frequency in European (non-Finnish) population (gnomAD: 353/126708)
Predicted benign in silico (SIFT: Tolerated (0.15); Polyphen22: benign (0.002))
Gene does not correlate with patient phenotype
ClinVAR status: Likely benign (RCV000296962.1)</td></tr>
<tr><td>NM_014956.4(CEP164):c.3869C>T, p.(Pro1290Leu)</td><td>Carrier of VUS</td><td>Rare variant in European (non-Finnish) population (10/126624, 0.00007897)
Equivocal in silico results (SIFT: Del (0); Polyphen22: Benign: 0.045)</td></tr>
</table>

Supplementary Data C

				Heterozygous change in recessive gene<br>Gene does not correlate with patient phenotype<br>Not previously reported as disease-causing
		NM_014336.3(AIPL1):c.737A>C, p.(Tyr246Ser) het	Carrier of VUS	Rare variant in European (non-Finnish) population (gnomAD: 21/1266694, 0.0001658)<br>Predicted deleterious *in silico* (SIFT: Deleterious (0.01); Polyphen2 (Probably damaging (0.991))<br>Highly conserved amino acid (GERP: 5.15)<br>Heterozygous change in recessive gene<br>Gene does not correlate with patient phenotype<br>Not previously reported as disease-causing
		NM_000554.4(CRX):c.196G>A, p.(Val66Ile) het	Benign	High frequency in European (non-Finnish) population (225/257790, 0.00724)<br>Equivocal *in silico* result (SIFT: Deleterious (0); Polyphen22: Benign (0.033))<br>Gene does not correlate with patient phenotype<br>ClinVAR status: Benign (RCV000297047.1)
		NM_001042472.2(ABHD12):c.787+3G>A, p.(splice) het	Carrier of VUS	Moderately high allele frequency in European (non-Finnish) population (34/24942, 0.001363)<br>Not predicted to affect splicing *in silico*<br>Heterozygous change in recessive gene<br>Gene does not correlate with patient phenotype<br>Not previously reported as disease-causing

Supplementary Data C

**B:II.2 Focused exome data filtering**				
**Filter**	**Number of Variants**			
**All**	8,935			
**All PAV**	1,439			
**Rare PAV**	199			
**Rare PAV, known IRD genes**	5	**Variants of Interest**	**Interpretation**	**Justification**
		NM_005559.3(LAMA1):c.2515C>T, p.(Pro839Ser) het	Carrier of VUS	High allele frequency in European (non-Finnish) population (gnomAD: 149/126698).<br>Predicted deleterious *in silico* (SIFT: Deleterious (0); Polyphen22: Probably damaging (1.0))<br>Heterozygous change in recessive gene.<br>Not previously reported as disease-causing
		NM_000186.3(CFH):c.350+1G>T, p.(splice) het	Likely Disease Causing	Mutation of canonical splice site.<br>Predicted to result in loss of donor splice-site *in silico*<br>Very rare allele in European (non-Finnish) population (gnomAD: 1/22296)<br>Consistent with retinal phenotype and mode of inheritance<br>Not previously reported as disease-causing
		NM_032119.3(ADGRV1):c.5785G>T, (p.Ala1929Ser) het	Benign	High allele frequency in European (non-Finnish) population (gnomAD: 173/126388).<br>ClinVAR status: Likely Benign (SCV000063285.4)<br>Equivocal *in silico* results (SIFT: Tolerated (0.16); Probably damaging 0.99)<br>Heterozygous change in recessive gene.<br>Gene does not correlate with patient phenotype
		NM_018942(HMX1):c.100C>A, p.(Arg34Ser) het	Benign	Absent from gnomAD<br>Predicted benign *in silico* (SIFT: Tolerated (0.76); Polyphen22: Benign (0))<br>Heterozygous change in recessive gene.

Supplementary Data C

				Gene does not correlate with patient phenotype.<br>Not previously reported as disease-causing
		NM_006702.4(PNPLA6):c.2945_2961del, p.(Arg983ProfsTer31) het	Benign	Absent from gnomAD<br>Heterozygous change in recessive gene.<br>Gene does not correlate with patient phenotype.<br>Not previously reported as disease-causing

**E:II.2 and E:III.11 WES data filtering**				
**Filter**	**Number of Variants**			
**Rare shared**	119			
**Rare shared PAV**	106			
**Rare PAV, known IRD genes**	4	**Variants of Interest**	**Interpretation**	**Justification**
		NM_000186.3(CFH):c.1291T>A, p.(Cys431Ser) het	Pathogenic mutation	Ultra-rare variant (MAF: 0.000004)<br>Predicted deleterious *in silico* (SIFT: Deleterious (0); Polyphen22 (1.0))<br>Same mutation reported as disease-causing on multiple occasions (Saunders *et al*., 2007; Dragon-Durey *et al*., 2003)
		ENST00000264448.6(ALMS1):c.1267G>A, p.(Val423Ile) het	Benign	High allele frequency (gnomAD: 783/276990)<br>Heterozygous change in recessive gene<br>Gene does not correlate with patient phenotype<br>Predicted benign in silico (SIFT: tolerated (<br>ClinVAR status: Benign (RCV000234139.2)

Supplementary Data C

		NM_178857.5(RP1L1):c.568C>T, p.(Arg190Cys) het	Benign	High allele frequency (gnomAD: 542/277010)<br>Equivocal *in silico* results (SIFT: Deleterious (0); Polyphen22: Benign (0.297))<br>ClinVAR status: Likely Benign (RCV000389130.1)<br>Gene does not correlate with patients phenotype<br>Not previously reported as disease-causing
		NM_001004334.3(GPR179):c.5563C>G, p.(Leu1855Val) het	Benign	High allele frequency (gnomAD: 725/276738)<br>Heterozygous change in recessive gene<br>Gene does not correlate with patient phenotype<br>Equivocal *in silico* results (SIFT: Tolerated (0.13); Polyphen22: Possibly damaging (0.657))<br>Not previously reported as disease-causing
