# Supplementary material for: Loss-of-Function Mutations in the CFH Gene Affecting Alternatively Encoded Factor H-like 1 Protein Cause Dominant Early-Onset Macular Drusen
Source: Ophthalmology. 2019 Oct;126(10):1410–21. doi: 10.1016/j.ophtha.2019.03.013 (PMC6856713; doi:10.1016/j.ophtha.2019.03.013)
Supplement: Supplemental Data D [file mmc7.pdf]

Supplementary Data D

Study	Study/Family ID	Age at onset (years)	Family history	Phenotype	Mutation	Domain	ExAC/gnomAD freq	Does mutation affect FHL-1
Boon et al., 2008	A	45-67	Yes	Confluent macular drusen, mid-peripheral cystoid drusen, chorioretinal atrophy	c.1222C>T; p.(Gln408Ter) het	7	0.000004073	Yes
Boon et al., 2008	B	48-68	Yes	Confluent macular drusen, chorioretinal atrophy, discrete pigmentary changes	c.1222C>T; p.(Gln408Ter) het	7	0.000004073	Yes
Boon et al., 2008	C	57	Yes	Large, confluent macular drusen, small peripheral drusen	c.3234G>T, p.(Arg1078Ser) het	18	17/246214 (0.00006905)	No
Boon et al., 2008	D	54	Yes	Large confluent macular drusen, mid-peripheral cystoid drusen, PED	c.350+6T>G het	int3_4	-	Yes
Boon et al., 2008	E	54	Yes	Confluent macular drusen, small mid-peripheral drusen, PED	c.1699A>G; p.(Arg567Gly) het	10	1/243758 (0.000004102)	No
Van de Ven et al., 2012	A	18-56	Yes	Extensive small and large confluent drusen, macula PED	c.550delA; p.(Ile184Leufs*33) het	3	-	Yes
Yu et al., 2014	II	<60	Yes	Early-onset AMD	c.269A>G; p.(Asp90Gly) het	2	1/246070 (0.000004064)	Yes
Yu et al., 2014	V	<60	Yes	Early-onset AMD	c.157C>T; p.(Arg53Cys) het	1	4/276658 (0.00001446)	Yes
Duvvari et al., 2015	1	n.d.	Unknown	Cuticular drusen	c.7C>G; p.(Leu3Val) het	SP	67/276914 (0.0002420)	Yes

Supplementary Data D

Duvvari et al., 2015	2	n.d.	Unknown	Cuticular drusen	c.428-2A>G	int2_3	-	Yes
Duvvari et al., 2015	3	n.d.	Unknown	Cuticular drusen	c.481G>T; p.(Ala161Ser) het	3	19/245854 (0.00007728)	Yes
Duvvari et al., 2015	5	n.d.	Unknown	Cuticular drusen	c.524G>A; p.(Arg175Gln) het	3	-	Yes
Duvvari et al., 2015	6	n.d.	Unknown	Cuticular drusen	c.578C>T; p.(Ser193Leu) het	3	-	Yes
Duvvari et al., 2015	7	n.d.	Unknown	Cuticular drusen	c.647T>C; p.(Ile216Thr) het	4	31/275272 (0.0001126)	Yes
Duvvari et al., 2015	8	n.d.	Unknown	Cuticular drusen	c.901_902del; p.(Ala301Asnfs*25) het	5	-	Yes
Duvvari et al., 2015	9	n.d.	Unknown	Cuticular drusen	c.1135T>C; p.(Trp379Arg) het	6	-	Yes
Duvvari et al., 2015	10	n.d.	Unknown	Cuticular drusen	c.1198C>A; p.(Gln400Lys) het	7	32/276144 (0 0001159)	Yes
Duvvari et al., 2015	11	n.d.	Unknown	Cuticular drusen	c.1198C>A; p.(Gln400Lys) het	7	32/276144 (0 0001159)	Yes
Duvvari et al., 2015	12	n.d.	Unknown	Cuticular drusen	c.2850G>C; p.(Gln950His) het	16	-	No
Duvvari et al., 2015	13	n.d.	Unknown	Cuticular drusen	c.2850G>C; p.(Gln950His) het	16	-	No
Duvvari et al., 2015	14	n.d.	Unknown	Cuticular drusen	c.2867C>T; p.(Thr956Met) het	16	358/277112 (0.001292)	No

Supplementary Data D

Duvvari et al., 2015	15	n.d.	Unknown	Cuticular drusen	c.3628C>T; p.(Arg1210Cys) het	20	40/276980 (0.0001444)	No
Duvvari et al., 2015	16	n.d.	Unknown	Cuticular drusen	c.3628C>T; p.(Arg1210Cys) het	20	40/276980 (0.0001444)	No
Hughes et al., 2016	Anon.	55	Unknown	Early-onset AMD	c.415C>G, p.(Pro139Ala) het	2	1/245274 (0.000004077)	Yes
Wagner et al., 2016	A	46-67	Yes	Large macular and extra-macular drusen in the region temporal to the macular	c.575G>T; p.(Cys192Phe) het	3	-	Yes
Wagner et al., 2016	B	n.d	Yes	Macular and exstensive extra-macular drusen (nasal to the macular and temporal to the vascular arcade)	c.790+1G>A	int4_5	-	Yes
Wagner et al., 2016	C	n.d	Yes	Early-onset AMD	c.524G>C; p.(Arg175Pro) het	3	1/245732 (0.000004069)	Yes
Wagner et al., 2016	D	n.d	Yes	Early-onset AMD	c.380G>A; p.(Arg127His) het	2	-	Yes
Duvvari et al., 2016	3	n.d.	No	Cuticular drusen	c.518C>G, p.(Ala173Gly) het	CC3	-	Yes

PED: Pigment epithelial detatchment; Pt: patient; Dx: diagnosed; AMD: Age-related macular degeneration; n.d.: not disclosed
